# Inhibition of triple negative breast cancer metastasis and invasiveness by novel drugs that target epithelial to mesenchymal transition

**DOI:** 10.1038/s41598-021-91344-7

**Published:** 2021-06-03

**Authors:** Elizabeth Garcia, Ismat Luna, Kaya L. Persad, Kate Agopsowicz, David A. Jay, Frederick G. West, Mary M. Hitt, Sujata Persad

**Affiliations:** 1 Department of Pediatrics, Faculty of Medicine and Dentistry, University of Alberta, 3-020R Katz Group Centre for Pharmacy and Health Research, Edmonton, AB T6G 2E1 Canada; 2 Department of Oncology, Faculty of Medicine and Dentistry, University of Alberta, Edmonton, Canada; 3 Department of Chemistry, Faculty of Science, University of Alberta, Edmonton, Canada

**Keywords:** Cancer, Drug discovery, Diseases, Oncology

## Abstract

Invasive breast cancer (BrCa) is predicted to affect 1 in 9 women in a lifetime;1 in 32 will die from this disease. The most aggressive forms of BrCa, basal-like/triple-negative phenotype (TNBC), are challenging to treat and result in higher mortality due high number of metastatic cases. There is a paucity of options for TNBC treatment, which highlights the need for additional innovative treatment approaches. NIH-III mice were injected in the abdominal mammary fat pad with luciferase-expressing derivative of the human TNBC cell line, MDA-MB-231 cells. Animals were gavage-fed with nitrofen at the doses of 1, 3 or 6 mg/kg/alternate days. However, several structural properties/components of nitrofen raise concerns, including its high lipophilicity (cLogP of nearly 5) and a potential toxophore in the form of a nitroarene group. Therefore, we developed analogues of nitrofen which lack the nitro group and/or have replaced the diaryl ether linker with a diarylamine that could allow modulation of polarity. In vitro anti-invasiveness activity of nitrofen analogues were evaluated by quantitative determination of invasion of MDA-MB-231-Luciferase cells through Matrigel using a Boyden chamber. Our in vivo data show that nitrofen efficiently blocks TNBC tumor metastasis. In vitro data suggest that this is not due to cytotoxicity, but rather is due to impairment of invasive capacity of the cells. Further, using an in vitro model of EMT, we show that nitrofen interferes with the process of EMT and promotes mesenchymal to epithelial transformation. In addition, we show that three of the nitrofen analogues significantly reduced invasive potential of TNBC cells, which may, at least partially, be attributed to the analogues’ ability to promote mesenchymal to epithelial-like transformation of TNBC cells. Our study shows that nitrofen, and more importantly its analogues, are significantly effective in limiting the invasive potential of TNBC cell lines with minimal cytotoxic effect. Further, we demonstrate that nitrofen its analogues, are very effective in reversing mesenchymal phenotype to a more epithelial-like phenotype. This may be significant for the treatment of patients with mesenchymal-TNBC tumor subtype who are well known to exhibit high resistance to chemotherapy.

## Introduction

Invasive breast cancer (BrCa) is a devastating disease that will affect 1 in 9 women in their lifetime^1^. 1 in 32 women will die from this disease^1^. In 2018, BrCa was estimated to account for 1 in 4 cancer cases diagnosed in women and accounted for the second largest amount of new cancer cases^2^. BrCa is the leading cause of mortality in women with cancer, and the 17th leading cause of death overall in the world^3,4^. BrCa is the most common type of cancer in 15–49 years old women, and the third most common type of cancer in women 50–59 years old. Invasive BrCas often display a basal-like phenotype, defined by expression of human epidermal growth factor receptor 2 (EGFR2 or HER2) and vimentin, and/or the triple-negative (TN) phenotype, which is negative for progesterone receptor, estrogen receptor, and HER2. Basal-like/triple-negative breast cancers (TNBC) comprise the most aggressive forms of BrCa with higher mortality due to a disproportionate number of metastatic disease cases^5,6^. 10–20% of all invasive breast cancer cases are TNBC^7^. TNBC, when compared to other BrCa subtypes, is more resistant to conventional therapy, which currently involves a combination of surgery, radiation therapy and neoadjuvant chemotherapy. Resistance may be related to reversion of germline BRCA1 mutations in TNBC which leads to reduction in sensitivity to DNA damaging agents, worsening patient prognosis^8^. Targeted therapies, an excellent approach to inhibiting tumor growth^9^, such as PARP inhibitors (Olaparib, Talazoparib) to induce synthetic lethality, have been recently approved by the US FDA for use in patients with deleterious (or suspect deleterious) germline BRCA mutation^10,11^. As an alternate approach, immunotherapy (Atezolizumab) in combination with chemotherapy (Paclitaxel) for unresectable locally advanced/metastatic PD-L1-positive TNBC is currently under investigation in several trials. However, the pathogenesis of TNBC is still poorly understood, and the mechanism(s) that drives these tumor cells to proliferate and metastasize remains unclear^6^. As a result, there is no standard targeted therapeutic regimen for the treatment of these types of cancer, which ultimately may contribute to the overall poor prognosis^6^. This paucity of options for TNBC treatment highlights the need for additional innovative treatment approaches.

Nitrofen is an herbicide which interferes with both oxidative and photosynthetic phosphorylation in the mitochondria and chloroplasts of plants^12^. Nitrofen was shown to have no adverse effects in adult rats upon exposure but induced multiple organ defects in embryos exposed at mid-gestation^12–16^, including congenital diaphragmatic herniation (CDH). The developmental toxicity displayed by nitrofen has been attributed to its effect in altering thyroid hormone status by binding receptors for T3^12–17^. Incidentally, many current chemotherapeutics (alkylating agents, topoisomerase inhibitors, histone deacetylase inhibitors) are also teratogens^18^. In adult animals nitrofen has been shown to be slightly toxic by oral, dermal and inhalation routes with an oral LD_50_ of 2.4–3.6 g/kg in rodents^12,16^. The no observable adverse effect level (NOAEL) of nitrofen for liver toxicity is 200 mg/kg/day^16^. Interestingly, nitrofen’s toxicity is much lower than many agents currently used for the treatment of BrCa which are associated with serious adverse health effects at therapeutic doses^19–21^. This project has grown out of an earlier study that examined the mechanism by which nitrofen induces developmental anomalies in rodent embryos. We proposed that nitrofen’s effects in developmental abnormalities may be mediated by perturbations in epithelial-mesenchymal transition (EMT), a key development component underlying organogenesis. By extrapolation, we hypothesized that if nitrofen reduces EMT, a process that has been implied to confer metastatic properties upon cancer cells by enhancing mobility, invasion, and resistance to apoptotic stimuli, then it might also have an impact on cancer metastasis. In this study we tested this hypothesis using a highly invasive/metastatic model of TNBC.

Using an in vitro Matrigel invasion assay as readout, we observed that TNBC cell lines (MDA-MB-468, MDA-MB-436, MDA-MB-231, MDA-MB-231-Luc, SUM 149) showed higher invasive potential than non-TNBC cell lines (MCF7, T47D, SKbr3), and that nitrofen treatment (1 & 10 μM) reduced invasive potential of TNBC lines to a greater extent than non-TNBC lines (Fig. S1). Further, we present compelling in vivo data which show that nitrofen efficiently blocks TNBC tumor metastasis, especially to the liver, following establishment of orthotopic xenografts of a luciferase-expressing derivative of the prototypical human TNBC cell line, MDA-MB-231 (MDA-MB-231-Luc) in nude mice. In vitro data suggest that this is not due to cytotoxicity, but rather is due to impairment of invasive capacity of the cells. Further, using an in vitro model of EMT, we show that nitrofen indeed interferes with the process of EMT and promotes mesenchymal to epithelial transformation. This may be a potential mechanism by which nitrofen affects the invasive potential TNBC cells.

However, several structural properties/components of nitrofen raise concerns regarding its putative efficacy as a therapeutic, including its high lipophilicity (cLogP of nearly 5)^22^ and a potential toxophore in the form of a nitroarene group. Therefore, we developed analogues of nitrofen which lack the nitro group and/or have replaced the diaryl ether linker with a diarylamine that could allow modulation of polarity. We show that three of the nitrofen analogues, significantly reduced invasive potential of TNBC, with two of them reducing invasive potential more efficiently compared to the parent compound, nitrofen. This inhibition of invasive potential may, at least partially, be attributed to the analogues’ ability to promote mesenchymal to epithelial-like transformation of TNBC cells.

## Materials and methods

### Cell lines and culture conditions

Cell lines used were: MCF7, T47D, SKBr3, MDA-MB-468, MDA-MB-436, SUM 149 and DU145 MDA-MB-231 (ATCC); MDA-MB-231-luc-D3H2LN (Caliper Life Sciences). The MDA-MB-231-luc-D3H2LN cell line was derived from a tumor isolated from the lymph node of a mouse after mammary fat pad injection with a luciferase-expressing MDA-MB-231 human breast cancer cell line^23^. STR profiling done on the MDA-MB-231-luc D3H2LN cell line matched MDA-MB-231 in the ATCC STR database. All cell lines were cultured in Dulbecco’s modified Eagle’s (DMEM) (Gibco), supplemented with 10% fetal bovine serum (FBS) (Gibco), 100 U/ml penicillin, 100 U/ml streptomycin (Gibco), 2 mM L-glutamine, incubated at 37ºC and 95% O_2_ / 5% CO_2._ Insulin 0.01 mg/ml (SIGMA) was added to medium for MCF7. Nitrofen treatment in vitro was carried out at 1 µM and 10 µM concentrations for 24 h in culture medium containing 1% FBS. This was the effective dose of nitrofen reported in two previous studies using cell culture assays without causing cell death^24,25^.

### Primary brain cortical cell culture

Rat cortical tissue was prepared from postnatal day 2 Long-Evan rats of either sex. Brains were dissected and cortices were removed from meninges and isolated and transferred to a Petri dish containing calcium- and magnesium-free (CMF) Hank’s Balanced Salt Solution (HBSS) (Gibco). Cortical tissues were enzymatically digested by 1 mg/mL papain (Thermo Scientific) for 10 min at 37 °C. DNase I (Millipore Sigma) was added to the digestion mix in the last 5 min of incubation. Fetal bovine serum (FBS) (Gibco) was added to stop the action of papain. Samples were centrifuged at 200 g for 1 min and supernatant was aspirated. Cortices were triturated by pipetting 10 times with a glass Pasteur pipette.

The cell suspension was filtered through a 70 µm Nylon mesh cell strainer with cell culture medium containing Neurobasal-A medium, supplemented with 2X B27, 4X glutaMAX I, and 2X Antibiotic–Antimycotic (Thermo Fisher Scientific Inc.). Cells were plated on poly-D-lysine coated wells at a density of 3 × 10^4^ cells/well in 24-well plates. Medium was changed 24 h after plating, and every 3 days thereafter. Treatment with nitrofen or analogues was started at day 7 in culture.

### TGF-β treatment EMT model

MCF7 BrCa cells were serum starved overnight and then treated with 2 ng/ml TGF-β in 0.2% BSA. Effect of TGF-β treatment was confirmed by monitoring of the constitutive phosphorylation of Smad 2/3 transcription factor.

### Animals and drug delivery

All the studies reported in this manuscript were conducted with the approval of the University of Alberta Health Sciences Animal Care and Use Committee in accordance with guidelines from the Canadian Council for Animal Care. We followed ARRIVE guidelines (as they apply to small animal study). We used a stratified randomization approach, and personnel who carried out bioluminescence imaging and monitored metastases was blinded to the treatment groups.

#### Tumor establishment

To establish single or bilateral orthotopic tumors, 2 × 10^6^ human MDA-MB-231-luc-D3H2LN cells (Caliper Life Science, Hopkinton, MA) were mixed 1:1 in Matrigel (Corning, Bedford, MA) to a total volume of 50 µL, which was then injected into the right (or both right and left) abdominal mammary fat pads of 6–8 week old female NIH-III mice (Charles River Laboratories). MDA-MB-231-luc-D3H2LN cells line was validated by STR profiling. Tumor sizes were measured twice weekly with calipers, and volumes calculated as described previously^26^. Mice with tumor volumes exceeding 1500 mm^3^ were euthanized.

#### Nitrofen treatment

Nitrofen (Sigma Aldrich Canada) was dissolved in olive oil and administered to anesthetized tumor bearing mice by oral gavage on alternate days at doses of 1, 3 or 6 mg/kg body weight. Treatment was initiated when tumors became palpable (~ 2–3 weeks post-tumor cell injection). Control animal were administered with olive oil vehicle by oral gavage.

#### In vivo bioluminescence

Bioluminescence imaging was carried out weekly to monitor metastases. Briefly, anesthetized mice were injected subcutaneously with 150 mg/kg of D-luciferin (Caliper Biosciences). Mice were then imaged with a Xenogen IVIS Spectrum 200 imaging system (Perkin Elmer, Waltham, MA, USA). For ex vivo luciferase detection, mice were injected with luciferin as above, then euthanized. Primary tumors, lungs, and livers were excised and bathed in a solution of luciferin then imaged.

### Synthesis and characterization of nitrofen analogues

Synthesis and characterization of nitrofen analogues are described in Supplemental Fig. 1 (Fig. S1). All starting materials and solvents were purchased from commercial suppliers and were used without further purification unless otherwise noted. Reactions were carried out in flame-dried glassware under nitrogen atmosphere using standard Schlenk technique unless otherwise stated. Transfer of anhydrous solvents and reagents was accomplished with oven-dried syringes. Thin layer chromatography was performed on glass plates precoated with 0.25 mm silica gel. Column chromatography was performed using 230 − 400 mesh silica gel. Samples were dissolved in CDCl3 to obtain nuclear magnetic resonance (NMR) spectra. Proton nuclear magnetic resonance spectra (1H NMR) were recorded at 500 MHz. Chemical shifts are given in ppm (parts per million) relative to residual CHCl3 (7.26 ppm) and coupling constants (J) are reported in hertz (Hz). Standard notation was used to describe the multiplicity of signals observed in 1H NMR spectra: broad (br), multiplet (m), singlet (s), doublet (d), triplet (t), etc. Carbon nuclear magnetic resonance spectra (13C NMR) were recorded at 125 MHz and are reported (ppm) relative to the center line of the triplet from chloroform-d (77.0 ppm). Infrared (IR) spectra were measured with a FT-IR 3000 spectrophotometer. Mass spectra were determined on a high-resolution electrospray positive ion mode spectrometer.

### Antibodies

Antibodies to E-cadherin (1:1000), N-cadherin (1:1000), phospho-Smad 2/3 (1:500), Smad (1:500), Phospho-Serine 473-AKT (1:1000), AKT (1:1000), and anti-vimentin (1:500) were from Cell Signaling (Beverly, MA, USA). Antibodies to ZO-1 (1:500), Twist (1:500), Snail (1:250) were from Abcam (USA). Antibody to β-actin (Santa Cruz) (1:5000), Anti-mouse IgG (1:5000), Anti-rabbit IgG (1:2000) were from GE Healthcare UK Ltd.

### Immunoblotting

Immunoblotting was done as described previously^27^. Cells were washed with PBS and lysed with 50 mMTris buffer (pH 8.0) containing 150 mM NaCl, 1% NP-40, 0.5% sodium deoxycholate, 1 mM phenylmethylsulphonyl fluoride, 5 μg/ml leupeptin and 25 μg/ml aprotinin. Protein concentration was quantified by bicinchoninic protein assay (Thermo Fisher Scientific Inc., Waltham, MA, U.S.A.). Proteins were separated by sodium dodecyl sulphate–polyacrylamide gel electrophoresis and transferred on to polyvinylidene difluoride (PVDF) membranes (Millipore, Billerica, MA, U.S.A.). PVDF membranes were blocked with 5% milk, probed with specific primary antibodies followed by peroxidase-conjugated secondary antibodies (GE Healthcare UK Ltd, Little Chalfont, U.K.) and visualized using Western Lightning® Plus-ECL (PerkinElmer, LAS Inc., Shelton, CT, U.S.A.) and x-ray developer (Fuji). In some instances, images were derived using Chemi DOC MP (Bio-Rad) and Image-Lab Touch Software (Bio-Rad). Densitometric analysis was performed by IMAGE J software (http://www.rsbweb.nih.gov/ij/). Histograms are representative of three or more independent experiments.

### Transwell® invasion assay

Cell invasion assay was carried out using Transwell® unit (8 µM) coated with BD Matrigel Basement Matrix was used (Corning, Bedford, MA). Cells were added at 5 × 10^4^ per invasion chamber and allowed to invade for 24 h at 37 °C and 95% O_2_ / 5% CO_2_ towards lower compartment with media containing 10% FBS. At completion of incubation period, invaded cells were fixed with ice-cold 100% methanol (−20 °C), stained with 0.5% crystal violet and number of invaded cells analyzed using 10X High Content Microscope and MetaExpress software.

### Alamar blue viability assay

The relative cytotoxicity of the nitrofen analogues was established using a primary rat brain cell culture and AlamarBlue assay. The AlamarBlue assay was carried out according to manufacturer’s instructions (ThermoFisher Scientific Inc.). Briefly, cells were incubated with 1 µM nitrofen or the analogues (A1, A5, A8) for 24 h in culture medium containing 1% FBS. Untreated cells were used as control. After 24 h incubation, AlamarBlue solution (10% [v/v] solution of AlamarBlue dye) in complete medium was added to each well. Wells containing only the AB solution/medium without cells was used as the blank. Following a 2 h incubation, AlamarBlue fluorescence was quantified at the excitation and emission wavelengths of 540 and 595 nm respectively, using a LUMIstar Omega reader. Percent viability was normalized to untreated cells: (sample relative fluorescent unit (RFU) − Blank) × 100/ (Untreated cells RFU- Blank).

### High content microscopy

High content microscopy was done as described previously^27^. Images were taken at 10X (NA 0.3) magnification using an automated, high content screening system, ImageXpress Micro XLS, Molecular Devices (USA). Briefly, a defined (3X3) number of images were taken per well and the resulted images were stored in a data storage server and analyzed using a predefined cell scoring algorithm in MetaXpress software package. that measures the blue staining in a bright field optical image on a per cell base, then averages the signal for the total population of cells.

### Statistics

A One-Way ANOVA, Dunnett test, n = 3 test (GraphPad PRISM Software; GraphPad Software, Inc., CA, USA) was used compare differences between groups. Results are presented as Mean ± SE and values.

## Results

### Nitrofen reduces in vitro invasive activity of breast cancer cells.

**Figure 1:**
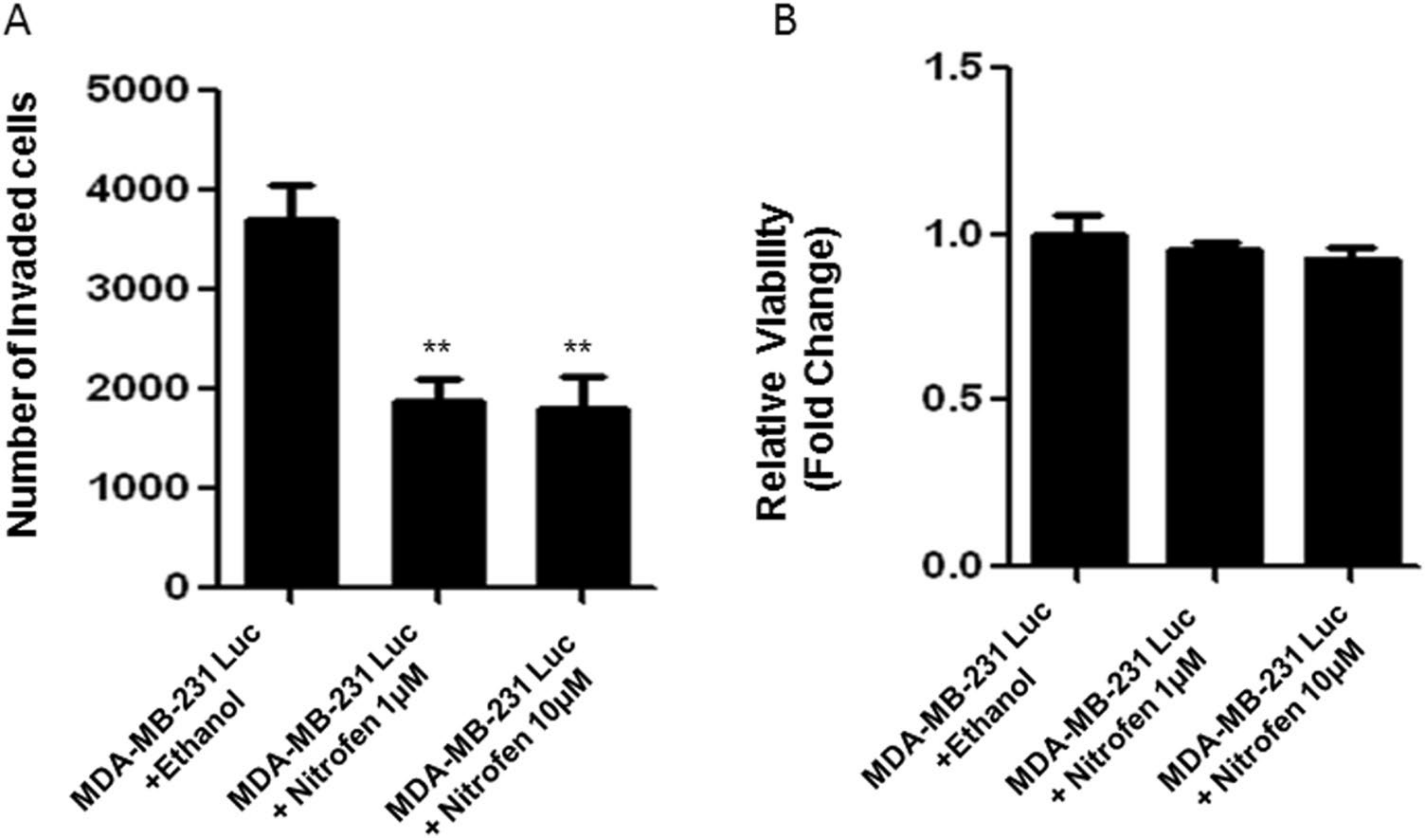
(**A**) Quantification of Matrigel invasion assay demonstrating that nitrofen (1 μM) significantly reduces the invasive potential of MDA-MB-231-Luc cells. Invasion assay was carried out using 40,000 cells/well, incubated for 24 h. Nitrofen was dissolved in Ethanol to prepare the stock concentration of 100 mM and diluted in DMSO to a final concentration of 1 uM and 10 uM. (**B**) AlamarBlue cell viability assay done under the same conditions shows that there is no change in relative viability of cells upon treatments. Data representative of 6 separate experiments each done in triplicate. **significantly altered from untreated control p < 0.01.

Using an in vitro Matrigel invasion assay, we observed that TNBC cell lines (MDA-MB-468, MDA-MB-436, MDA-MB-231, SUM 149) showed higher invasive potential than non-TNBC cell lines (MCF7, T47D, SKbr3). Further, nitrofen treatment (1 & 10 μM) reduced invasive potential of TNBC lines to a greater extent than non-TNBC lines (Fig. S1). We further determined the effect of nitrofen on the invasive potential of the luciferase expressing MDA-MB-231 (MDA-MB-231-luc-D3H2LN) cell line (MDA-MD-231-Luc). Accordingly, we used MDA-MB-231-luc-D3H2LN (MDA-MB-231-Luc) cells to carry out in vitro cell invasion assays in the presence and absence of 1 and 10 μM nitrofen. We observed significant reduction in invasive potential of MDA-MB-231-Luc cells in the presence of 1 and 10 μM nitrofen relative to untreated control (Fig. 1A). AlamarBlue assay done under the same conditions shows that there is no change in relative viability of cells upon any treatments (Fig. 1B).

### Nitrofen reduces in vivo metastasis of MDA-MB-231-luc-D3H2LN breast cancer cells

**Figure 2:**
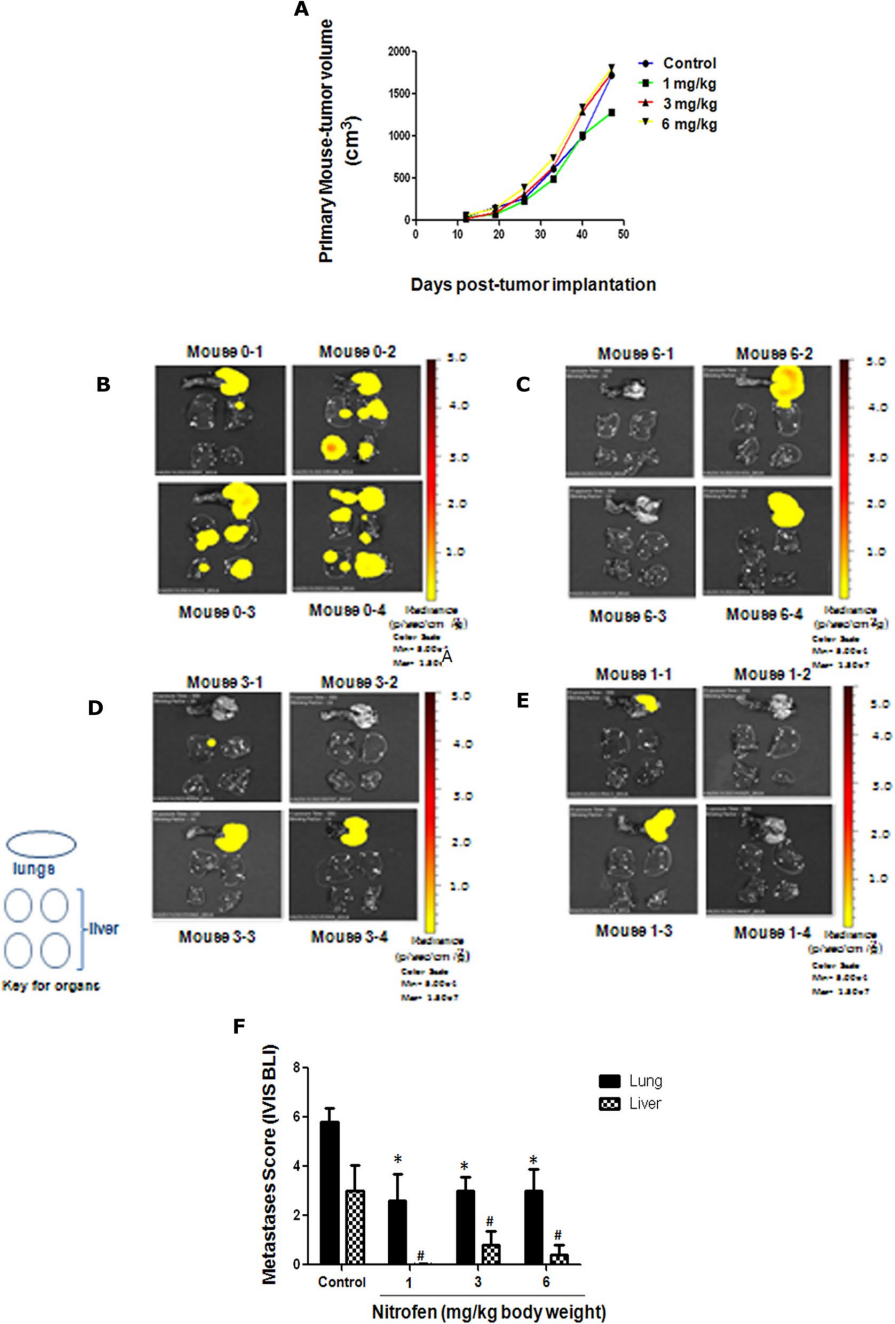
(**A**) Growth of primary tumors following treatment with nitrofen. Mice with established bilateral orthotopic 231-Luc tumors were administered the following agents by oral gavage on alternate days for 6 weeks, starting on day 12: olive oil (control) or nitrofen at 1, 3, or 6 mg/kg body weight as indicated. (**B-E**) Bioluminescence ‘heat map’ of metastases in the lungs and liver of tumor-bearing animals from this experiment. (**B**) untreated (control (fed with olive oil): Mouse 0–1 to 0–4); (**C**) Nitrofen at 6 mg/kg body Wt: Mouse 6–1 to 6–4; (**D**) Nitrofen at 3 mg/kg body Wt: Mouse 3–1 to 3–4; (**E**) Nitrofen at 1 mg/kg body Wt: Mouse 1–1 to 1–4. (**F**) Metastasis score determined by quantification of IVIS bioluminescence (shown in panels **B**–**E**) in the lungs and liver of untreated animals (control) or treated with various doses of nitrofen (1, 3, 6 mg/kg body Wt) every alternate day for 6 weeks. n = 5–10 tumor bearing mice per group. * significantly different compared to control lungs P < 0.05, # significantly different compared to control liver P < 0.05.

We used an orthotopic tumor model with MDA-MB-231-luc-D3H2LN (MDA-MB-231-Luc) cells to allow in vivo tracking of tumor burden and metastasis in NIH-III nude mice. Mice were treated with low doses of nitrofen (1, 3 and 6 mg/kg/day, alternate days) or vehicle (olive oil) for 6 weeks. Mice were monitored for primary tumor growth and were examined weekly for metastasis by in vivo bioluminescence imaging. At 8 weeks post-tumor-implantation, tissues were examined by *ex-vivo* bioluminescence imaging. BrCa is known to metastasize preferentially to the lungs, liver and bone. Fig. S2 shows quantifiable bioluminescence ‘heat maps’ of MDA-MB-231-Luc tumors in abdominal mammary fat pads and at metastatic sites by in vivo imaging and demonstrated extensive metastases in untreated mice by 8 weeks post-tumor implantation (Fig. S2). Nitrofen had no significant effect on primary tumor growth (Fig. 2A). Body weight of mice remained statistically comparable under the different treatment conditions through the duration of the treatments (Fig. S3), suggesting there were no significant adverse effects of the treatments on the mice.

Ex vivo bioluminescence imaging at end point, showed extensive bioluminescence signal fluxes in both lungs and liver of untreated mice (Fig. 2B). However, 6 weeks of oral gavage treatment with nitrofen, at the doses of 6 mg/kg (Fig. 2C), 3 mg/kg (Fig. 2D) or 1 mg/kg (Fig. 2E) body weight on alternate days, significantly reduced metastatic growth in the lungs and liver. Quantification of the number of metastases (metastatic score) using ex vivo IVIS bioluminescence flux analysis shows significant decrease of metastases in both lungs and liver upon treatment of tumor bearing mice with all doses of nitrofen (1, 3 and 6 mg/kg body weight) (Fig. 2F).

### Nitrofen inhibits epithelial to mesenchymal transition

We have previously observed, in an unrelated study, that nitrofen induces perturbation in epithelial-mesenchymal transition (EMT) using a PTEN-knockdown model of prostate cancer cell lines (Fig. S4). Knockdown of PTEN resulted in EMT alterations that were significantly attenuated in the presence of 1 μM and 10 μM nitrofen (Fig. S4).

**Figure 3:**
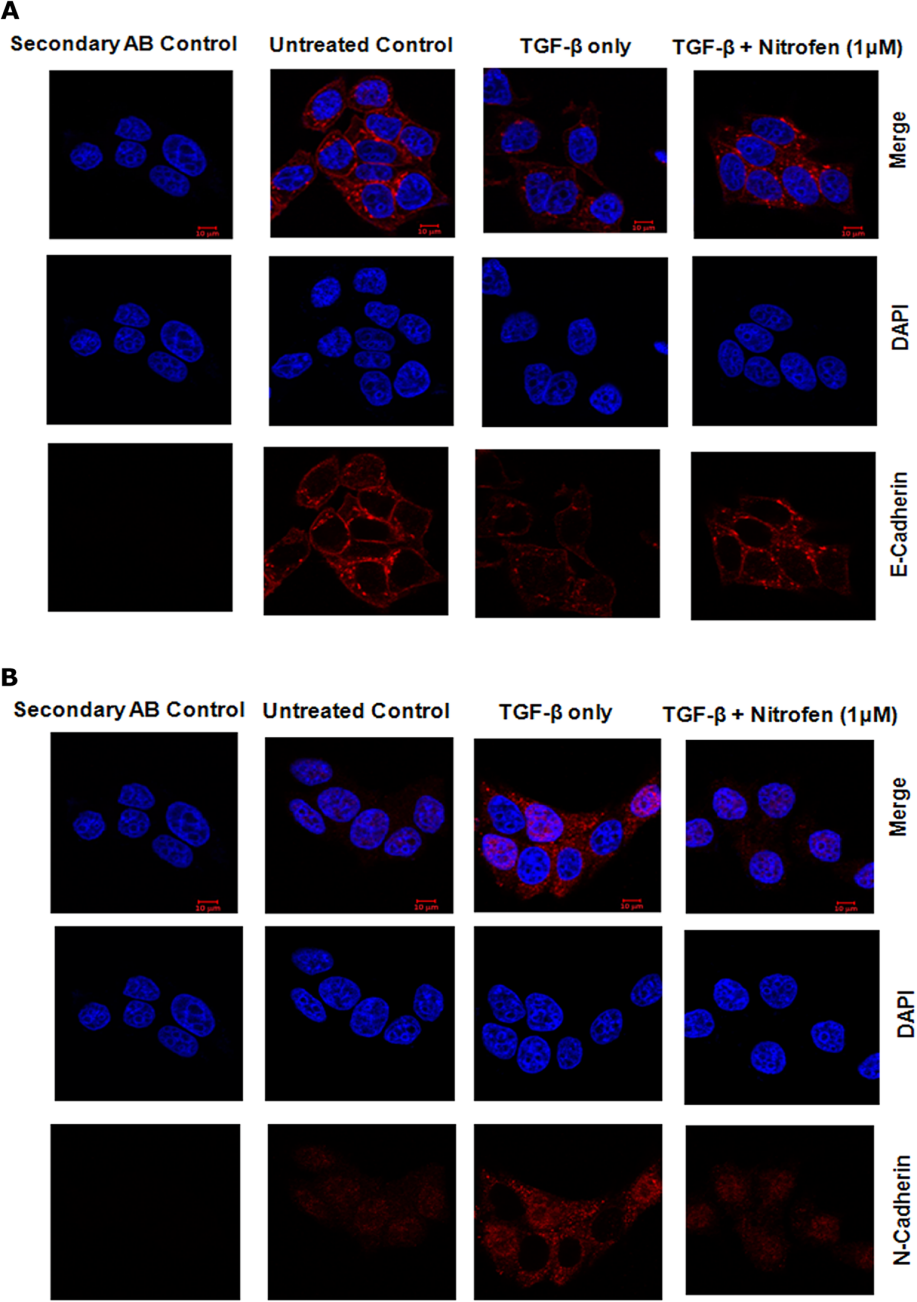

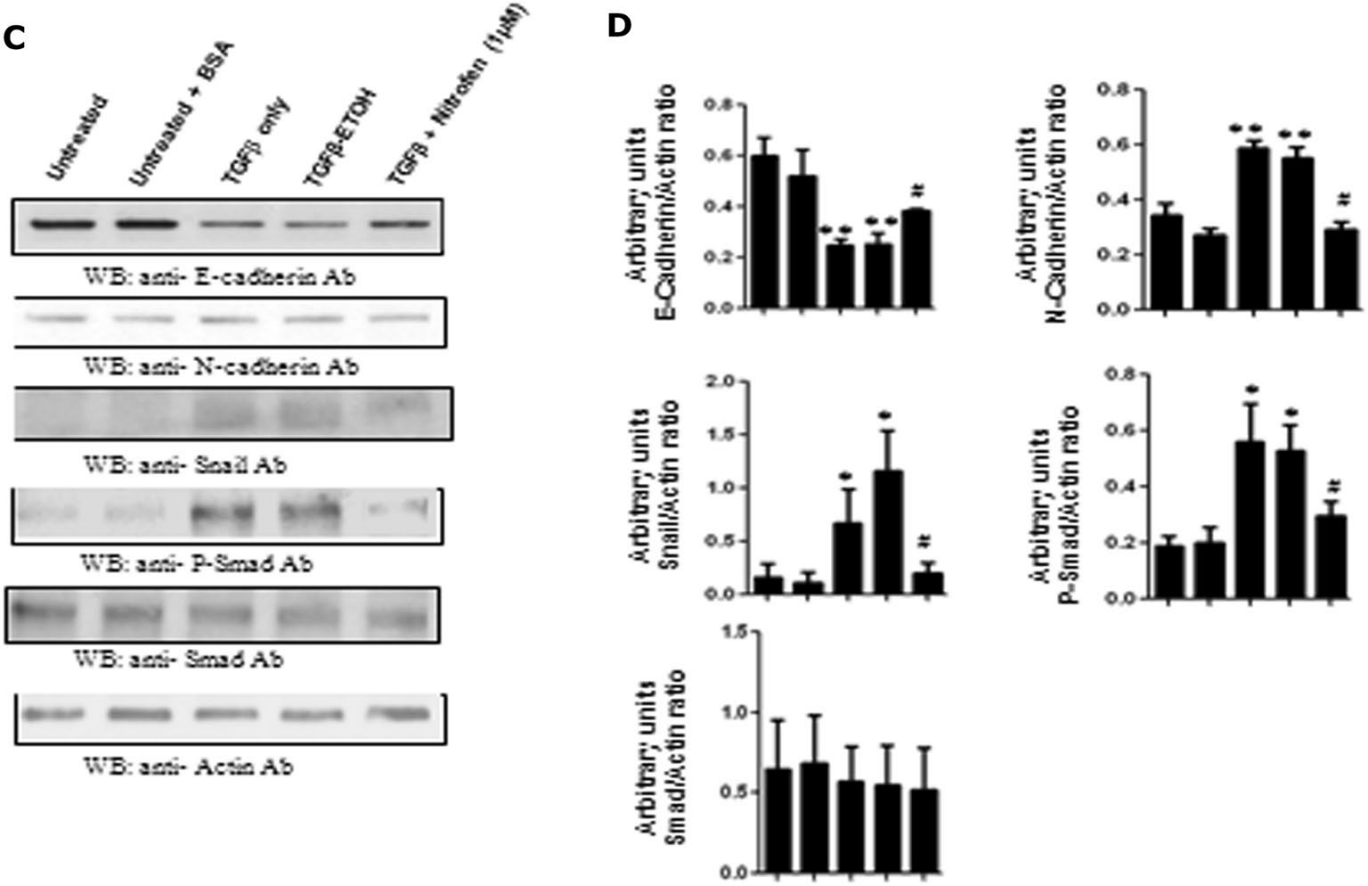
TGF-β-induced EMT changes in MCF-7 breast cancer cells is prevented in the presence of nitrofen (1 μM) for 24 h: (**A**) TGF-β treatment caused significant decrease in E-cadherin levels in MCF-7 breast cancer cells which was prevented in the presence of nitrofen (1 μM). (**B**) TGF-β treatment caused significant increase in N-cadherin levels in MCF-7 breast cancer cells which was prevented in the presence of nitrofen (1 μM). (**C**) Western blot analysis shows that there was a significant down-regulation of the epithelial protein E-cadherin and up-regulation of the mesenchymal proteins N-cadherin and snail upon treatment with TGF-β. These alterations were prevented in the presence of nitrofen (1 μM). Western blot of phospho-Smad (p-Smad) and Smad proteins upon treatment with TGF-β show significant increase in p-Smad levels compared to untreated control. This increase was prevented in the presence of nitrofen (1 μM). Data representative of 6 separate experiments each done in triplicate. * p < 0.01 significantly different from untreated control; #p < 0.01 significantly different from TGF-β treated.

In the present study, we induced EMT in the MCF7 breast cancer cell line by treatment of these cells with 2 ng/ml TGF-β. Immunofluorescence analysis of the cells shows that while treatment with TGF-β resulted in a down-regulation of epithelial marker E-cadherin and up-regulation of the mesenchymal marker N-cadherin levels (Fig. 3A,B), these alterations were significantly prevented in the presence of 1 μM nitrofen (Fig. 3A,B). This is confirmed by immunoblot analysis which shows down-regulation of the epithelial marker E-cadherin and up-regulation of the mesenchymal markers N-cadherin and Snail upon treatment with TGF-β (Fig. 3C,D). These alterations were significantly reversed/prevented in the presence of 1 μM nitrofen (Fig. 3C,D). Treatment with TGF-β increased phosphorylation of Smad 2/3, which was significantly lower in the presence of 1 μM nitrofen (Fig. 3C,D). These results suggest that nitrofen effectively reduced EMT alterations induced by treatment with TGF-β.

### Synthesis of nitrofen analogues

**Figure 4:**
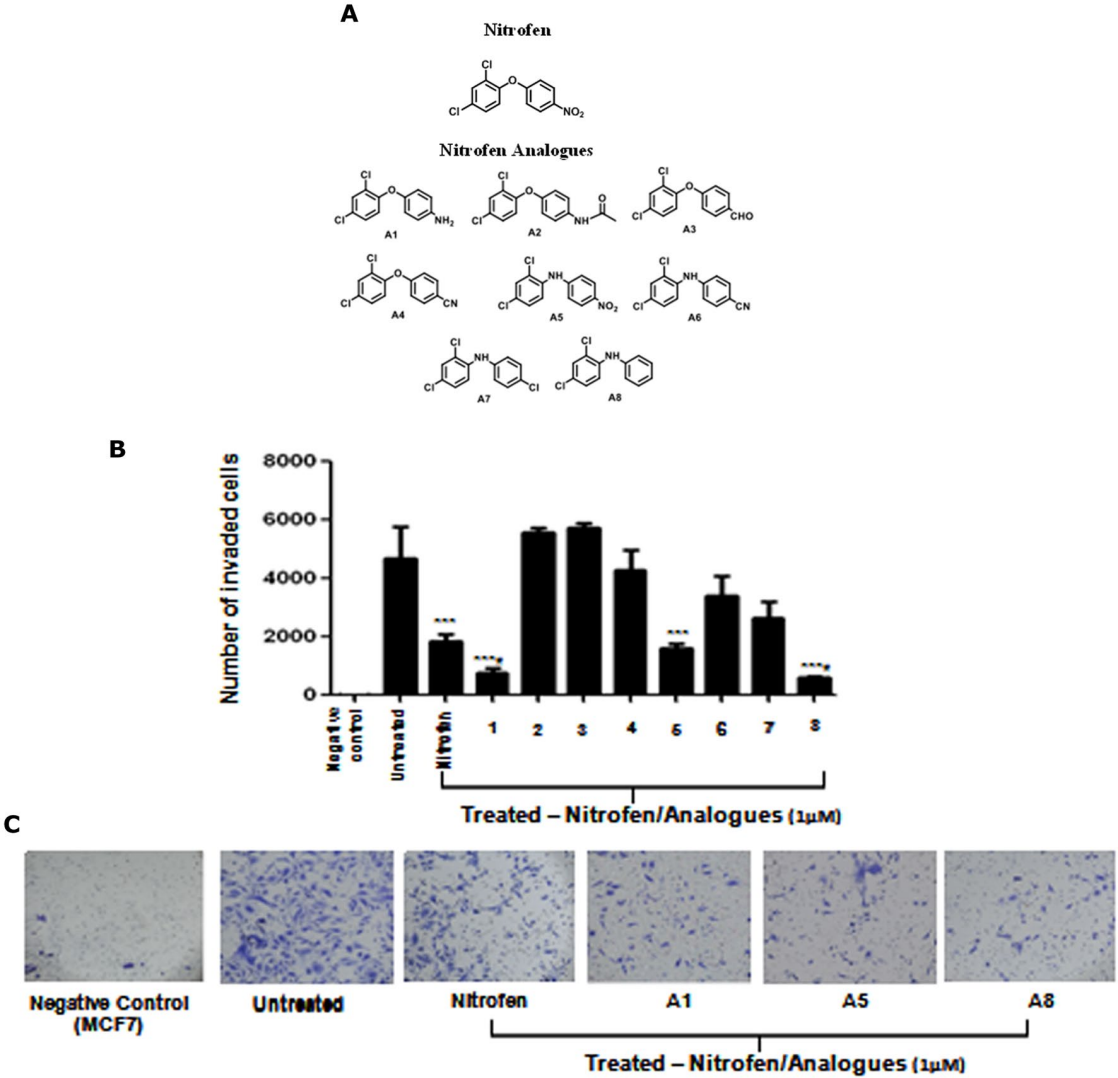

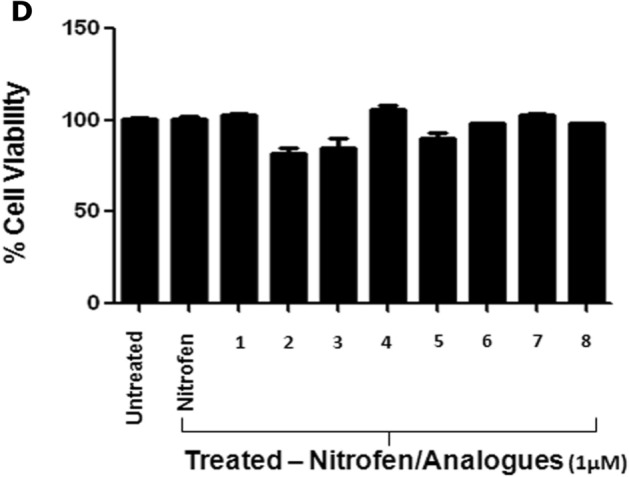
(**A**) Nitrofen analogues (A1 -A8) generated from the parental nitrofen which lack the nitro group and/or have replaced the diaryl ether group with a diarylamine. (**B**) Quantification of Matrigel invasion assay of MDA-MB-231-Luc cells treated with nitrofen and nitrofen analogues (A1–A8), same conditions as Fig. 3. Data shows that analogues A1, A5 and A8, significantly reduce the invasive potential of MDA-MB-231-Luc cells. Analogues A1 and A8 reduce the invasive potential of MDA-MB-231-Luc cells to a greater extent compared to nitrofen. (**C**) Photomicrograph of invading MDA-MB-231-Luc cells in the presence or absence of treatment with nitrofen and analogues A1, A5 and A8. (**D**) AlamarBlue cell viability assay done under the same conditions shows that there is no change in relative viability of cells with treatments. Non-invasive MCF-7 cells were used as a negative control. Data representative of 6 separate experiments each done in triplicate. *significantly altered from untreated control p < 0.05; # significantly altered from nitrofen-treated cells p < 0.05.

Although nitrofen showed anti-invasion activity both in vitro and in vivo, there is no compelling reason to believe that its structure is optimized for the as-yet-unknown cellular target. We chose to make several modifications to probe the importance of various structural elements of nitrofen, especially those that raise concerns, such as the nitroarene (a potential toxophore) and the relatively nonpolar diaryl ether. (Note that nitrofen has a cLogP value of nearly 5.) Thus, we prepared eight analogues (A1–A8; Fig. 4A) in which the nitro group was replaced with a reduced aniline or acetanilide, other electron-withdrawing groups (chloride, aldehyde or nitrile), or removed entirely. Furthermore, for several derivatives the diaryl ether was replaced with a less lipophilic diarylamine. These simple changes were designed to obtain a preliminary assessment of the extent to which the scaffold could be perturbed, which would be useful in the eventual design of probe molecules to identify the protein target of nitrofen, but we also imagined that some of these compounds might display superior properties to the parent.

### Nitrofen analogues reduce in vitro invasive activity of breast cancer cells

**Figure 5:**
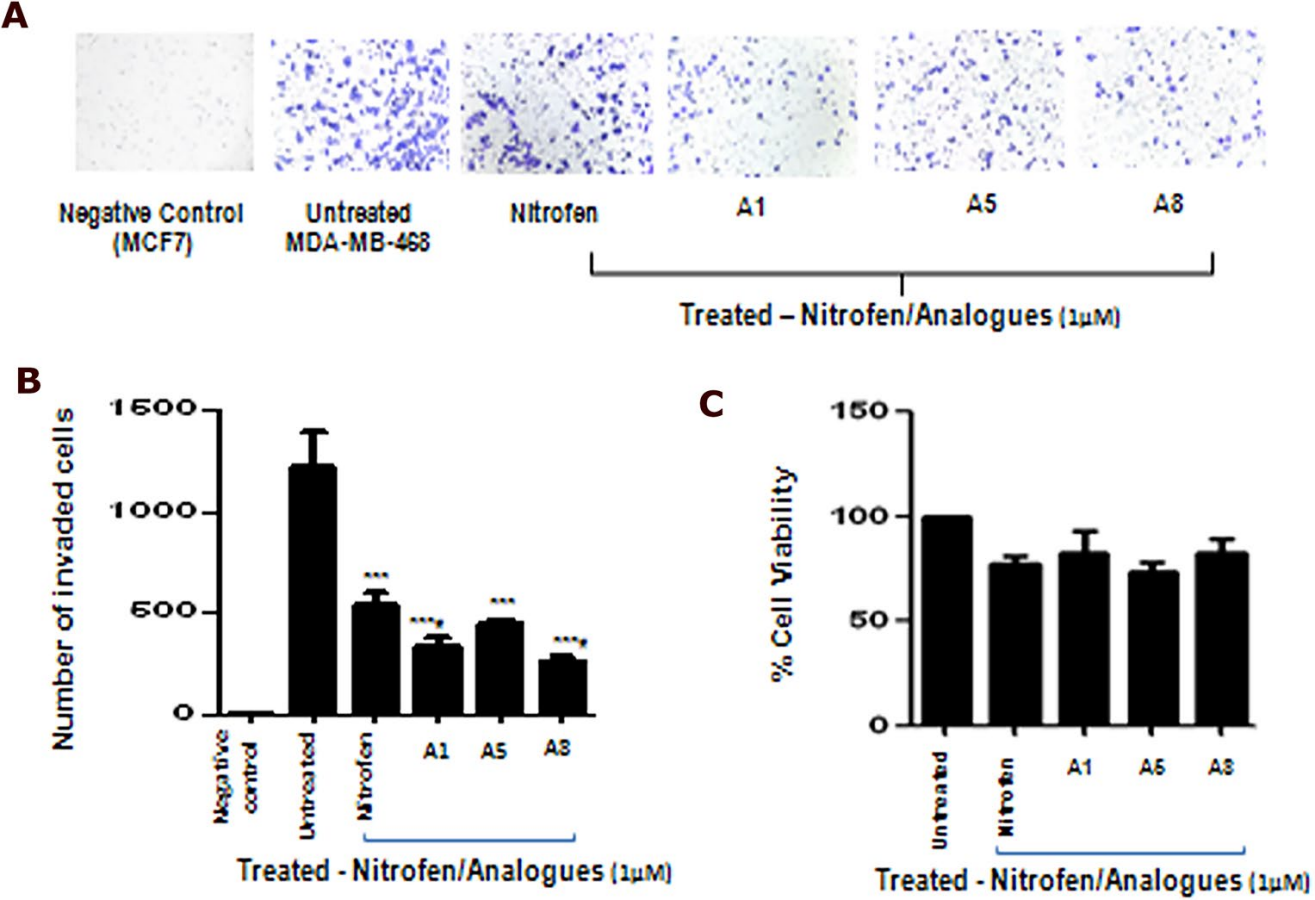
(**A**) Photomicrograph of invading MDA-MB-468 TNBC cells in the presence or absence of treatment with nitrofen and analogues A1, A5 and A8. (**B**) Quantification of Matrigel invasion assay of MDA-MB-468 cells treated with nitrofen and nitrofen analogues A1, A5 and A8. Data show that analogues A1, A5 and A8 significantly reduce the invasive potential of MDA-MB-468 cells. Analogues A1 and A8 reduce the invasive potential of MDA-MB-468 cells to a greater extent compared to nitrofen. (**C**) AlamarBlue cell viability assay done under the same conditions shows that there is no change in relative viability of cells with treatments. Non-invasive MCF-7 cells were used as a negative control. Data representative of 6 separate experiments each done in triplicate. *significantly altered from untreated control p < 0.05; # significantly altered from nitrofen-treated cells p < 0.05.

We examined the efficacy of 1 µM concentrations of all the analogues (Fig. 4A) on in vitro invasive potential of MDA-MB-231-Luc cells (Fig. 4B). Our results show that 1 µM concentrations of 3 of the 8 analogues (A1, A5, A8) significantly reduced invasive potential of MDA-MB-231-Luc cells relative to untreated control, and 2 of them (A1 & A8) were superior to the parent compound nitrofen in reducing the invasive potential of these cells (Fig. 4B,C). AlamarBlue assay under the same conditions show that there was no alteration in the relative viability of treated cells compared to untreated cells (Fig. 4D). Similar efficacy of nitrofen analogues (A1, A5, A8) in inhibiting invasive potential was also observed in another Basal B TNBC cell line MDA-MB-436 (Fig. S6). We also examined whether nitrofen analogues (A1, A5, A8) inhibited invasive potential of the Basal A TNBC cell line MDA-MB-468. Our results show that 1 µM concentrations of 3 of the 8 analogues (A1, A5, A8) significantly reduced invasive potential of MDA-MB-468 cells with no significant alterations in relative viability of cells (Fig. 5A–C).

**Figure 6:**
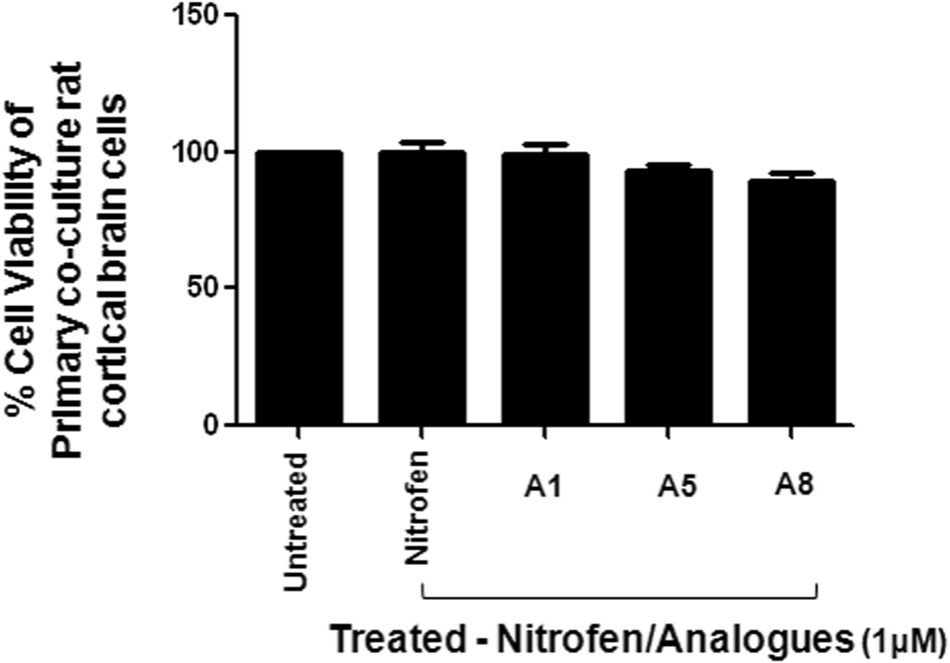
AlamarBlue cell viability assay of primary culture of rat cortical brain cells treated with nitrofen and nitrofen analogues (A1, A5, and A8). Data show that there is no change in relative viability of cells with any treatments. Data representative of 6 separate experiments each done in triplicate.

We further assessed potential cytotoxic effects of nitrofen and the three effective analogues (A1, A5, A8) using primary rat brain culture of mixed brain cells (neurons, astrocytes, microglia) and the Live/Dead assay. Our results show that treatment with 1 μM nitrofen or analogues (A1, A5, A8) was not cytotoxic to these primary rat brain cortical cell cultures (Fig. 6).

### Nitrofen and its analogues induce mesenchymal to epithelial type transition (MET) in mesenchymal TNBC cell lines

**Figure 7:**
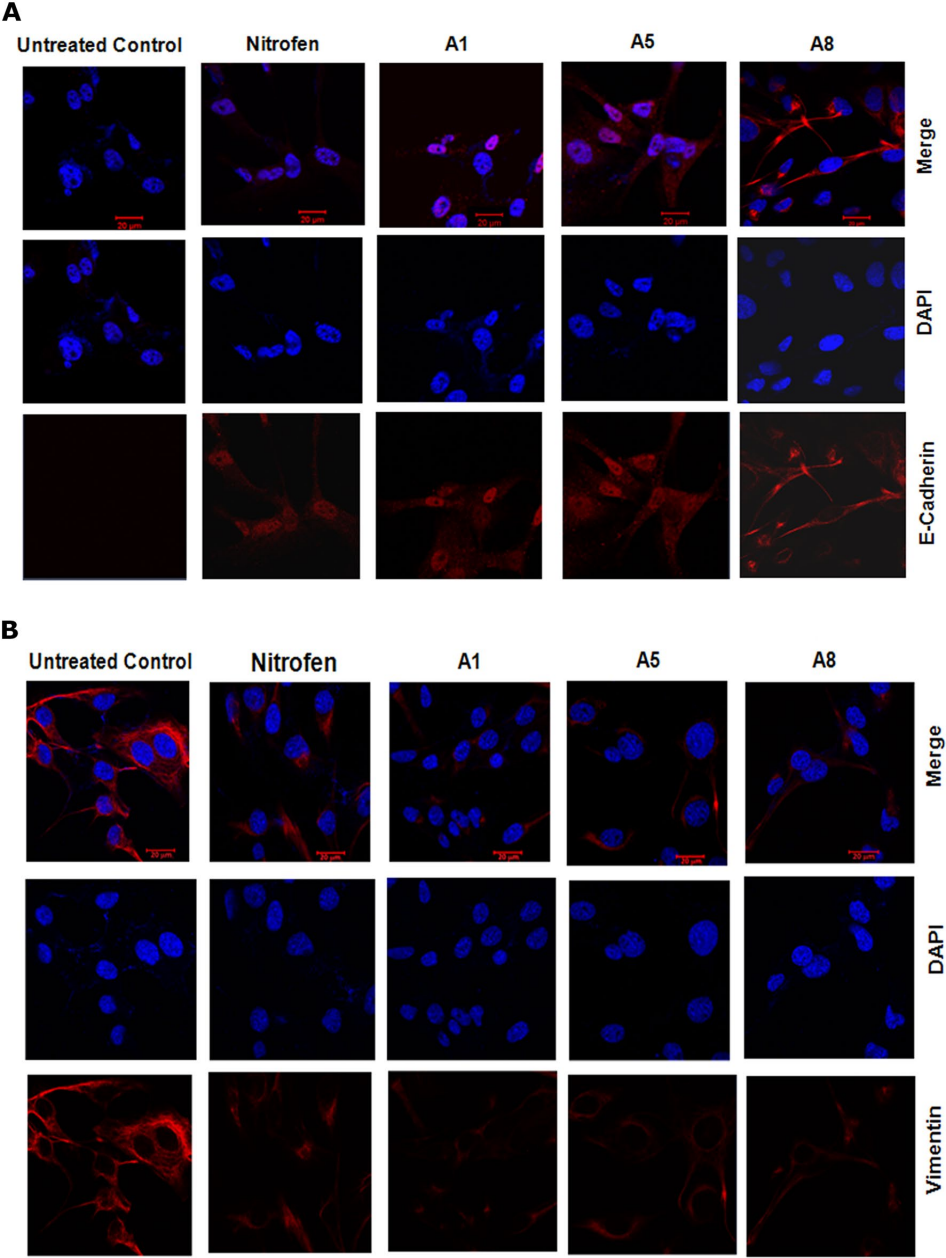

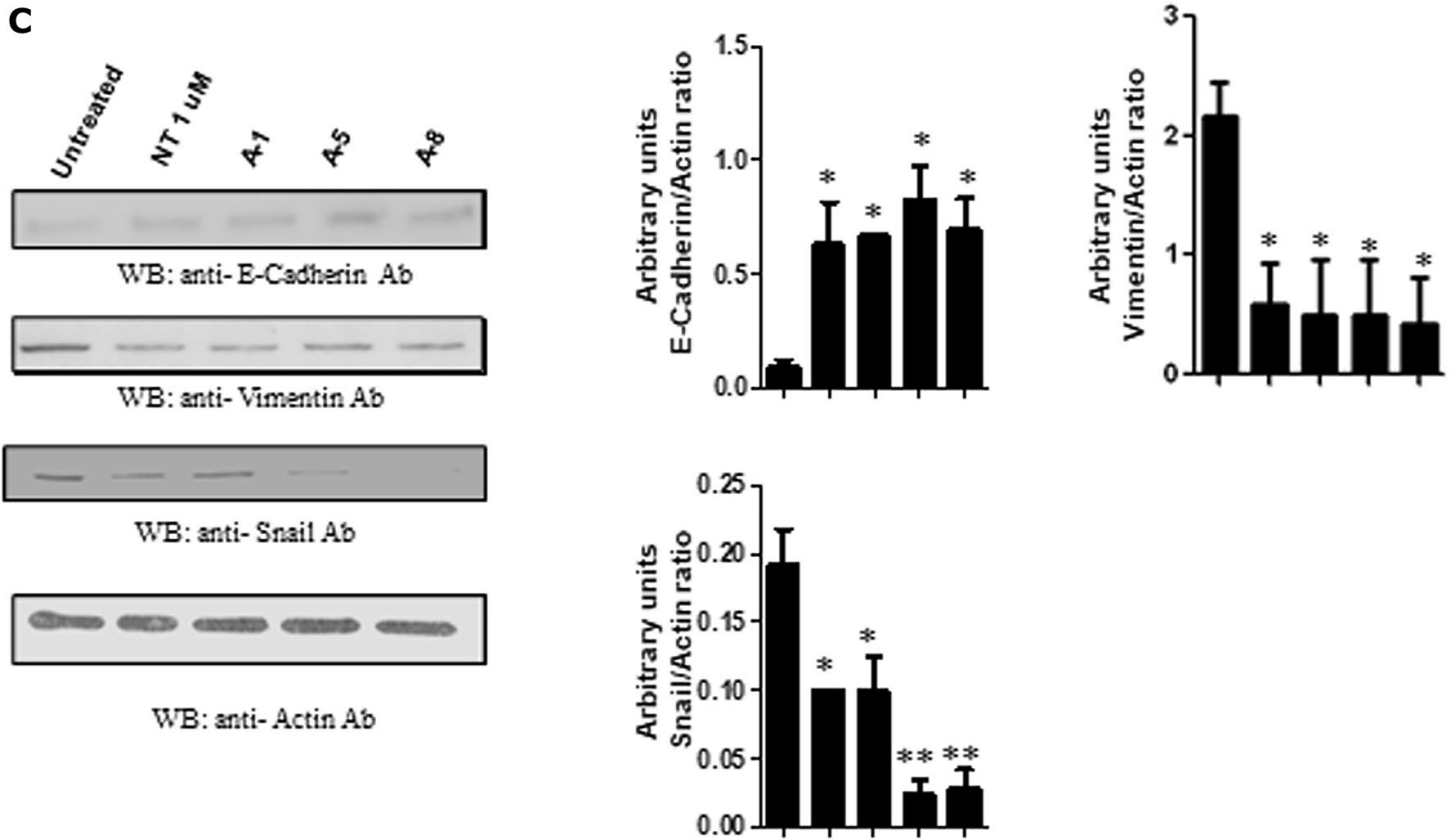
Nitrofen analogues promote mesenchymal to epithelial transformation in MDA-MB-231-Luc TNBC cell line. (**A**) Treatment with nitrofen and analogues A1, A5 and A8 at 1 μM concentration for 24 h results in the appearance of epithelial marker E-cadherin in MDA-MB-231-Luc TNBC cell line which is mesenchymal-like in phenotype. (**B**) Treatment with nitrofen and analogues A1, A5 and A8 results in significant decrease in cellular expression of vimentin protein in MDA-MB-231-Luc TNBC cell line. Data representative of 6 separate experiments each done in triplicate. (**C**) Western blot analysis shows that treatment with nitrofen and analogues A1, A5 and A8 at 1 μM concentration for 24 h results in the appearance of epithelial marker E-cadherin in MDA-MB-231-Luc cell line. Treatment with nitrofen and analogues A1, A5 and A8 results in significant decrease in cellular expression of mesenchymal markers vimentin, twist and snail. Data representative of 6 separate experiments each done in triplicate. *significantly altered from untreated control p < 0.05; ** significantly altered from untreated control p < 0.01.

Mesenchymal-like TNBC is a subgroup of TNBC that harbor mesenchymal-like features such as enriched expression of genes involved in EMT. It is recognized that elevated vimentin and decreased E-cadherin protein levels are characteristic of mesenchymal-like TNBC subgroup. MDA-MB 231 cells do not express E-cadherin but do express copious levels of vimentin protein. MDA-MB-231 cells do not express N-cadherin. Treatment with nitrofen (1 μM) or nitrofen analogues A1, A5 and A8 (1 μM) resulted in the appearance of E-cadherin (Fig. 7A) in these cells and significant down-regulation of vimentin protein (Fig. 7B) reminiscent of a mesenchymal to epithelial like transition. MET alterations were further confirmed by immunoblot analysis for epithelial marker E-cadherin which was significantly upregulated and mesenchymal marker vimentin, and Snail (Fig. 7C) which were significantly down-regulated, upon treatment with nitrofen analogues (A1, A5, A8).

Similar results were seen upon treatment of another mesenchymal cell line MDA-MB-436 (Fig. S7). It should be pointed out that we immunoblotted for the epithelial marker ZO-1 in the MDA-MB-436 cells as we were unable to detect any expression of E-cadherin protein in these cells in the presence and absence of treatments. This is likely because the CDH1 (E-cadherin gene) is known to be highly methylated in this cell line and they do not express any mRNA for E-cadherin.

## Discussion

Invasive breast cancer is a devastating disease, the most aggressive forms of which is the basal-like/triple-negative phenotype (TNBC). TNBC does not express estrogen receptors or progesterone receptors and lacks HER2 amplification. Patients diagnosed with TNBC have a higher risk of disease relapse within 5 years than patients treated for other breast cancer subtypes. This highlights the need for innovative treatment approaches. This study tested a novel pharmacological approach to inhibiting invasion and metastasis of TNBC in vitro and in an in vivo mouse model. This avenue of research is derived from an unrelated earlier study in our lab examining the mechanism of action by which the compound nitrofen induces known developmental anomalies in rodent embryos. We speculated that nitrofen’s effects in developmental abnormalities may, at least partially, be related to a perturbation in EMT, a key development component underlying organogenesis. Indeed, using PTEN-knockdown in DU145 prostate cancer cells as a model for EMT we have shown that nitrofen in fact does interfere with the process of EMT (Fig. S4): knockdown of PTEN resulted in down-regulation of E-cadherin and up-regulation of N-cadherin in DU145 prostate cancer cells (Fig. S4). These alterations were significantly attenuated in the presence of 1 µM and 10 µM nitrofen. Knockdown of PTEN in DU145 cells also resulted in an increase in the levels of the transcription factors Snail and Twist which were significantly prevented in the presence of 1 µM and 10 µM nitrofen (Fig. S4). In the present study, we tested this hypothesis using TGFβ-induced EMT in the MCF7 breast cancer cell line. Treatment of MCF7 cells with TGFβ resulted in down-regulation of E-cadherin and up-regulation of N-cadherin. These alterations were significantly attenuated in the presence of 1 µM nitrofen. By extrapolation, we hypothesized that if nitrofen perturbs EMT, then it may have an impact on cancer metastasis and therefore could be a putative starting point in developing an effective new drug for treating metastatic BrCa where EMT is a critical component of the pathogenesis.

We present provocative in vivo data which shows that nitrofen (2,4-dichloro-4’-nitrodiphenyl ether) efficiently blocks metastatic tumor growth, following establishment of orthotopic xenografts in nude mice of a luciferase-expressing derivative of the prototypical human TNBC cell line MDA-MB-231. MDA-MB-231 cells undergo spontaneous metastasis via unconfirmed mechanisms although microRNA, especially miR-21, is thought to play a role^28,29^. Our results show that while nitrofen had little effect on the primary tumor weight or body weight, the level of metastases was appreciably lower in the lungs and liver of mice treated with 6, 3 or 1 mg/kg/day nitrofen. It is notable that the effect of nitrofen treatment is more robust in the livers of the treated animals than in the lungs. This may be due to the oral mode of administration of nitrofen and the effectively greater concentrations of the drug in the liver. Evaluation of the effects of nitrofen on invasive potential of a panel of BrCa cell lines as well as the MDA-MB-231-Luc cell line using an in vitro Matrigel invasion assay, showed that nitrofen significantly attenuates the invasive potential of BrCa cell lines, with a significantly greater inhibitory effect on the invasive potential of TNBC cell lines compared to the non-TNBC lines. These results corroborated our observations in the in vivo model, suggesting that nitrofen’s effect in altering invasive potential is, at least partially, responsible for the observed attenuation of MDA-MB-231-Luc metastasis in the in vivo mouse model.

Cumulatively, these data suggest that nitrofen is effective in decreasing invasive potential of breast cancer cells in vitro*,* effectively blocking metastasis in vivo, which may have significant implications in blocking/limiting BrCa metastasis in patients.

We also developed analogues of nitrofen designed to have reduced lipophilicity and lacking the aromatic nitro group that may undergo conversion to toxic metabolites. The aim was to generate feasible structurally modified nitrofen analogues that retain anti-invasive activity with potentially superior pharmacokinetic properties and reduced long-term toxicity. Here we report synthesis of 8 analogues of nitrofen, all of which lack the nitro group and/or have replaced the diaryl ether group with a diarylamine. 3 of the 8 compounds significantly reduced invasive potential of two TNBC cell lines, MDA-MB-231-Luc and MDA-MB-468, in vitro relative to untreated control, and 2 of them (A1 & A8) were superior to the parent compound nitrofen. Further, nitrofen and the three analogues were completely nontoxic to both cell lines at the concentration that caused a greater than twofold decrease in invasive potential compared to untreated controls. Importantly, treatment of a very vulnerable primary culture of normal cortical brain cells with nitrofen or its analogues (A1, A5, A8) did not result in any cytotoxicity. This is very significant, as these observations stand in contrast to the cytotoxic effects on normal cell lines that are frequently seen with many chemotherapeutic agents.

TNBC is a heterogeneous disease based on gene expression signatures, biological properties and clinical outcome. TNBC can be separated into 6 TNBC subtypes displaying unique GE and ontologies, including 2 basal-like (BL1 and BL2), an immunomodulatory (IM), a mesenchymal (M), a mesenchymal stem–like (MSL), and a luminal androgen receptor (LAR) subtype^30^. TNBC-BL exhibits the highest responsiveness to chemotherapy^31^, including platinum-based chemotherapy, that is directed at targeting the DNA-repair deficiency^32–35^. Immune based therapies, including immune-checkpoint blockade^36,37^ and tumor vaccines^38,39^, are being actively developed to treat the “immunomodulatory” subtype of TNBC. Luminal/apocrine TNBC with androgen receptor overexpression and HER-2-enriched TNBC overlap significantly with the other three subgroups and clinical trials are currently underway to test combinatorial therapies using AR inhibitors for the luminal/apocrine TNBC group and HER2 targeted therapies for the HER2-enriched TNBC^40–42^. For mesenchymal-like TNBC (ML-TNBC), a cancer stem cell profile and expression of mesenchymal markers are highly correlated to chemotherapy resistance^43^. These tumors are very difficult to treat although many promising treatments targeted at components of pathways that promote EMT (MAPK or Wnt pathways) are under investigation^44–49^. Our data show that nitrofen and its analogues very effectively reverse the mesenchymal-like phenotype of the TNBC cell lines to a more epithelial phenotype. We postulate that this is, at least partially, responsible for the effectiveness of these drugs in inhibiting in vivo metastasis of TNBC tumors and in vitro invasive potential of TNBC cell lines. However, the exact EMT-promoting pathway that is targeted by this group of drugs is not known at present and is currently under investigation.

Our cumulative data show that nitrofen, and more importantly its analogues that lack the potentially toxic aromatic nitro group, are significantly effective in limiting the invasive potential of TNBC cell lines with minimal cytotoxic effect to normal cells. Although we have demonstrated that this group of compounds are very effective in reversing mesenchymal phenotype to a more epithelial-like phenotype, determination of their molecular target(s) is paramount to deciphering the putative clinical relevance of this treatment. Importantly, nitrofen analogues potentially could be of benefit in combinatorial treatment with other chemotherapeutics for patients with mesenchymal-TNBC tumor subtype who are well known to exhibit high resistance to chemotherapy. However, it should be pointed out that in addition to the potential and very beneficial effect on the mesenchymal (Basal B) subgroup of TNBC, nitrofen analogues likely have cellular effects in addition to inducing MET. This is evident by the ability of these compounds in inhibiting the invasive potential of the epithelial (Basal A) TNBC cell line MDA-MB-468. This suggests that nitrofen analogues have a broader spectrum of efficacy in the treatment of TNBC as they are effective in treating both epithelial and mesenchymal subgroups of TNBC, albeit via different mechanisms.

In conclusion, this study demonstrates the efficacy of nitrofen in preclinical models, but more importantly, it highlights the efficacy of its analogues in blocking invasive properties of TNBC and sheds light on a putative mechanism for this effect. Further work must be carried out to determine the exact pathways by which these drugs suppress TNBC invasiveness/metastasis. This will inform the future development of the selected nitrofen analogues into a clinically usable form and the development of novel treatment strategies for metastatic BrCa, with high potential for clinical translation.

### Ethical approval and consent to participate

All animal experiments were approved by the Health Sciences Animal Care and Use Committee at University of Alberta in compliance with guidelines established by the Canadian Council on Animal Care (protocol #AUP00000251).

### Consent for publication

The submission of this manuscript has been approved by all authors.

### Compliance with ARRIVE guidelines

We followed ARRIVE guidelines (as they apply to small animal study). We used a stratified randomization approach, and personnel who carried out bioluminescence imaging and monitored metastases was blinded to the treatment groups.

## Supplementary Information

Supplementary Information.
