# Supplementary material for: Inhibition of triple negative breast cancer metastasis and invasiveness by novel drugs that target epithelial to mesenchymal transition
Source: Sci Rep. 2021 Jun 3;11:11757. doi: 10.1038/s41598-021-91344-7 (PMC8175347; doi:10.1038/s41598-021-91344-7)
Supplement: Supplementary file 1 — Supplementary Information. [file 41598_2021_91344_MOESM1_ESM.pdf]

Inhibition of triple negative breast cancer metastasis/invasiveness by novel drugs that target epithelial to mesenchymal transition

Elizabeth Garcia [ξ#], Ismat Luna[ψ#], Kaya L Persad[ξ], Kate Agopsowicz[ʅ], David A Jay[ξ], Frederick G West[ψ], Mary M Hitt[ʅ], Sujata Persad[ξ*]

Electronic Supplementary Material: experimental procedures and characterization data for chemical synthesis of nitrofen analogues **A1**–**A8**.

Table of Contents

General Experimental Information: **S-1**

Procedures and Physical Data for A1–A8: **S-2–S-6**

References: **S-6–S-7**

Proton and Carbon NMR Spectra for A1–A8: **S-8–S-15**

General Experimental Information. All starting materials and solvents were purchased from commercial suppliers and were used without further purification unless otherwise noted. Reactions were carried out in flame-dried glassware under nitrogen atmosphere using standard Schlenk technique unless otherwise stated. Transfer of anhydrous solvents and reagents was accomplished with oven-dried syringes. Thin layer chromatography was performed on glass plates precoated with 0.25 mm silica gel. Column chromatography was performed using 230−400 mesh silica gel. Samples were dissolved in $CDCl_3$ to obtain nuclear magnetic resonance (NMR) spectra. Proton nuclear magnetic resonance spectra (^{1}H NMR) were recorded at 500 MHz. Chemical shifts are given in ppm (parts per million) relative to residual $CHCl_3$ (7.26 ppm) and coupling constants (*J*) are reported in hertz (Hz). Standard notation was used to describe the multiplicity of signals observed in ^{1}H NMR spectra: broad (br), multiplet (m), singlet (s), doublet (d), triplet (t), etc. Carbon nuclear magnetic resonance spectra (^{13}C NMR) were recorded at 125 MHz and are reported (ppm) relative to the center line of the triplet from chloroform-d (77.0 ppm). Infrared (IR) spectra were measured with a FT-IR 3000 spectrophotometer. Mass spectra were determined on a high-resolution electrospray positive ion mode spectrometer.

Synthesis of Analogues A1–A8.

A) Hydrogenation followed by amidation reaction:

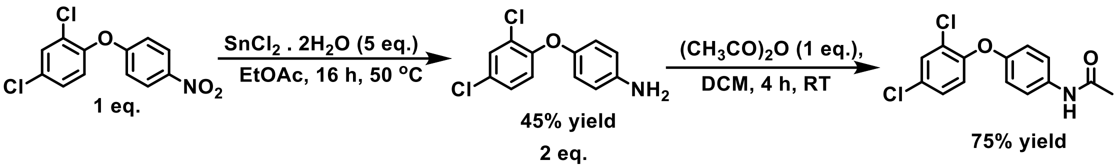

B) S_NAr reaction:

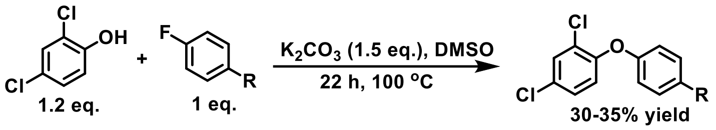

C) Buchwald-Hartwig coupling reaction:

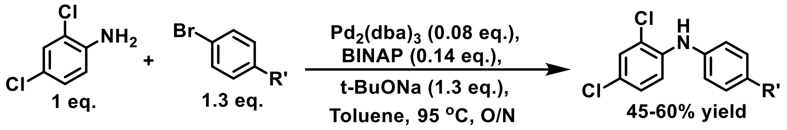

Figure S1. Synthetic routes to analogues A1-A8: reduction/acylation of nitro group, formation of diaryl ether via nucleophilic aromatic substitution, or palladium-catalyzed aromatic amination via Buchwald-Hartwig coupling reaction.

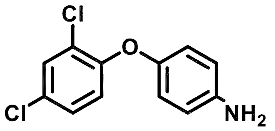

4-(2,4-Dichlorophenoxy)aniline (analogue A1). Known compound **A1** was synthesized using the method available in literature [1]. 2,4-Dichloro-1-(4-nitrophenoxy)benzene (1.0 mmol, 0.28 g, 1.0 equiv) was dissolved in 10 mL of EtOAc, and then $SnCl_2.2H_2O$ (5.0 mmol, 1.13g, 5.0 equiv) was added into the solution. The reaction mixture was stirred at 50 °C for 16 h. Completion of the reaction was monitored by TLC with 20% EtOAc/Hexane eluent system. The reaction mixture was partitioned between EtOAc and sat. $NaHCO_3$. The organic layer was collected, and the aqueous layer was extracted with EtOAc (2 x 50 mL). The organic layers were combined, washed with sat. $NaHCO_3$, brine, dried with Na_2SO_4, filtered and concentrated. Crude material was purified by column chromatography (gradient elution with 5% to 10% EtOAc/hexanes) to obtain compound **A1** as brown solid (1.3 g, 52% yield); IR (cast film) ν_{max} = 3444, 3377, 3213, 3091, 3043, 1623, 1582, 1573, 1507, 1472, 1389, 1321, 1255, 1244, 875, 824, 782 cm^{-1}; ^1H NMR (500 MHz, $CDCl_3$) δ 7.42 (d, *J* = 2.6 Hz, 1H), 7.10 (dd, *J* = 8.8, 2.5 Hz, 1H), 6.83 (app d, *J* = 8.8 Hz,

2H), 6.75 (d, J = 8.8 Hz, 1H), 6.67 (app d, J = 8.9 Hz, 2H), 3.61 (br s, 1H); ^{13}C NMR (125 MHz, $CDCl_3$) δ 153.2, 148.0, 143.2, 130.2, 127.7, 127.5, 124.9 (2C), 120.5, 118.8, 116.2 (2C); HRMS (ESI) m/z calcd for $C_{12}H_{10}Cl_2NO$ $[M + H]^+$ 254.0134; found 254.0139.

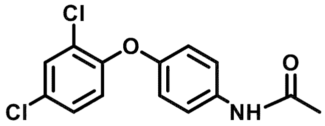

A2

2,4-Dichloro-1-(4-acetamidophenoxy)benzene (analogue A2). Synthesis of compound **A2** was carried out through amidation rection between analogue **A1** and acetic anhydride according to the reported procedures [2]. 4-(2,4-dichlorophenoxy)benzenamine (1.9 mmol, 0.48 g, 1.0 equiv) was dissolved dry DCM (5 mL) acetic anhydride (2.3 mmol, 0.2 mL, 1.2 equiv) was then added to the solution and the reaction was stirred at room temperature for around 3 hours and monitored by TLC. Upon completion, the reaction mixture was washed with a saturated solution of Na_2CO_3, the organic layers dried with Na_2SO_4 and the solvent removed under reduced pressure. The product was obtained in quantitative yield (1.4g, 75% yield) as yellowish solid; IR (cast film) ν_{max} = 3258, 3199, 3136, 3061, 1664, 1618, 1560, 1538, 1505, 1471, 1277, 1254, 1196, 1098, 825, 699 cm^{-1}; ^{1}H NMR (500 MHz, $CDCl_3$) δ 7.48-7.54 (m, 3H), 7.17 (dd, J = 6.3, 2.4 Hz, 1H), 7.10 (br s, 1H), 6.9 (app d, J = 8.9 Hz, 2H), 6.87 (d, J = 8.7 Hz, 1H); ^{13}C NMR (125 MHz, $CDCl_3$) δ 168.1, 152.9, 151.7, 133.8, 130.5, 128.9, 127.9, 126.2, 121.7 (2C), 120.8, 118.8 (2C), 24.5; HRMS (ESI) m/z calcd for $C_{14}H_{12}C_{l2}NO_2$ $[M + H]^+$ 296.0240; found 296.0253.

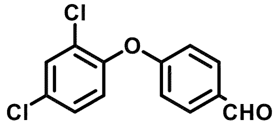

A3

4-(2,4-Dichlorophenoxy)benzaldehyde (analogue A3). Synthesis of compound **A3** was carried out by employing S_NAr reaction according to the reported procedure with minor modifications [3]. To a solution of 4-fluorobenzaldehyde (1.0 mmol, 0.1 mL, 1.0 equiv) and 2,4-dichlorophenol (1.2 mmol, 0.19 g, 1.2 equiv) in DMSO (1 mL), K_2CO_3 (0.15 mmol, 0.21 g) was added. The mixture was then heated to 100 °C for 2 h. TLC confirmed the completion of reaction. The reaction mixture was then poured into iced water (100 mL) which caused formation of precipitation. Then the solid was collected by filtration. The filtered cake was washed with water, and then dried over under

reduced pressure. Further purification was not required. Compound **A3** was obtained as off-white solid (0.8 g) with 30% yield; IR (cast film) ν_{max} = 3082, 2832, 2741, 1693, 1603, 1579, 1502, 1472, 1425, 1301, 1258, 1214, 1157, 1099, 1057, 853, 829, 787, 728 cm^{-1}; ^{1}H NMR (500 MHz, $CDCl_3$) δ 9.93 (s, 1H), 7.86 (app d, *J* = 8.8 Hz, 2H), 7.52 (d, *J* = 2.5 Hz, 1H), 7.30 (dd, *J* = 6.1, 2.6 Hz, 1H), 7.08 (d, *J* = 8.7 Hz, 1H), 7.00 (app d, *J* = 8.5 Hz, 2H); ^{13}C NMR (125 MHz, $CDCl_3$) δ 190.6, 162.1, 149.3, 132.0 (2C), 131.8, 131.2, 130.9, 128.5, 127.9, 123.5, 116.8 (2C); HRMS (EI) m/z calcd for $C_{13}H_8Cl_2O_2$ $[M]^+$ 265.9901; found 265.9903.

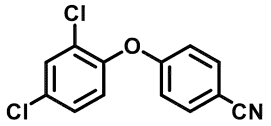

A4

4-(2,4-Dichlorophenoxy)benzonitrile (analogue A4). The method above for **A3** was employed to synthesize compound **A4** with the following stoichiometric amounts: 4-fluorobenzonitrile (1.0 mmol, 0.12 g, 1.0 equiv); 2,4-dichlorophenol (1.2 mmol, 0.19 g, 1.2 equiv) and K_2CO_3 (0.15 mmol, 0.21 g) to afford compound **A4** as brownish solid in 35% yield; IR (cast film) ν_{max} = 3094, 3079, 2227, 1606, 1581, 1501, 1472, 1415, 1382, 1296, 1256, 1238, 1166, 1099, 1057, 1014, 833, 809, 753, 708, 677, 661 cm^{-1}; ^{1}H NMR (500 MHz, $CDCl_3$) δ 7.62 (app d, *J* = 8.8 Hz, 2H), 7.51 (d, *J* = 2.5 Hz, 1H), 7.30 (dd, *J* = 6.2, 2.5 Hz, 1H), 7.06 (d, *J* = 8.7 Hz, 1H), 6.94 (app d, *J* = 8.8 Hz, 2H); ^{13}C NMR (125 MHz, $CDCl_3$) δ 160.5, 148.9, 134.3 (2C), 131.5, 130.9, 128.6, 123.5, 118.5, 117.1 (2C), 106.6; HRMS (EI) m/z calcd for $C_{13}H_7Cl_2NO$ $[M]^+$ 262.9905; found 262.9907.

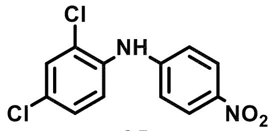

A5

2,4-Dichloro-N-(4-nitrophenyl)-benzenamine (analogue A5). Synthesis of compound **A5** was carried out by employing Buchwald-Hartwig cross coupling reaction according to the reported procedure with minor modifications [3]. 2,4-Dichlorobenzenamine (1.0 mmol, 0.16 g), 1-bromo-4-nitrobenzene (1.3 mmol, 0.26 g), $Pd_2(dba)_3$ [tris(dibenzylideneacetone)dipalladium(0)] (0.08 mmol, 0.07 g), BINAP (2,2′-bis(diphenylphosphino)-1,1′-binaphthyl) (0.14 mmol, 0.08 g), and sodium tert-butoxide (1.3 mmol, 0.12 g) were dissolved in 10 mL of toluene and stired at 95 °C under overnight reflux. The course of the reaction was followed by TLC. On cooling to room

temperature, the reaction mixture was partitioned between 1.0 M aqueous sodium bisulphate and diethylether. The diethylether phase was washed once with saturated sodium bicarbonate and brine, dried over $MgSO_4$, filtered and concentrated. Crude product was purified by column chromatography (gradient elution with 5% to 10% EtOAc/hexanes) to obtain compound **A5** as brown solid (0.15 g, 53% yield); IR (cast film) ν_{max} = 3355, 3086, 3064, 1604, 1583, 1535, 1484, 1471, 1424, 1380, 1340, 1326, 1114, 1101, 1052, 856, 840, 819, 796, 747, 703, 688, 675, 655 cm^{-1}; ^{1}H NMR (500 MHz, $CDCl_3$) δ 8.17 (app d, *J* = 9.2 Hz, 2H), 7.47 (d, *J* = 2.4 Hz, 1H), 7.38 (d, *J* = 8.7 Hz, 1H), 7.25 (dd, *J* = 5.6, 3.0 Hz, 2H), 7.02 (app d, *J* = 9.1 Hz, 2H); ^{13}C NMR (125 MHz, $CDCl_3$) δ 148.2, 141.2, 135.6, 130.1 (2C), 128.9, 127.9, 126.2, 126.1, 121.4, 115.4 (2C); HRMS (ESI) m/z calcd for $C_{12}H_7C_{l2}N_2O_2$ $[M - H]^-$ 280.9890; found 280.9889.

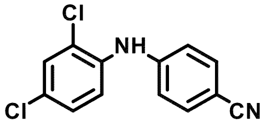

A6

4-[(2,4-Dichlorophenyl)amino]-benzonitrile (analogue A6). The method above for **A5** was employed to synthesize compound **A6** with the following stoichiometric amounts: 2,4-dichlorobenzenamine (1.0 mmol, 0.16 g), 1-bromo-4-benzonitrile (1.3 mmol, 0.28 g), $Pd_2(dba)_3$ [tris(dibenzylideneacetone)dipalladium(0)] (0.08 mmol, 0.07 g), BINAP (2,2′-bis(diphenylphosphino)-1,1′-binaphthyl) (0.14 mmol, 0.08 g) and sodium tert-butoxide (1.3 mmol, 0.12 g) to afford compound **A6** as brownish solid (0.12 g) in 50% yield; IR (cast film) ν_{max} = 3330, 3070, 2220, 1609, 1589, 1516, 1466, 1417, 1327, 1226, 1176, 1101, 868, 825, 767, 746, 694, 665 cm^{-1}; ^{1}H NMR (500 MHz, $CDCl_3$) δ 7.55 (app d, *J* = 4.9 Hz, 2H), 7.45 (d, *J* = 2.4 Hz, 1H), 7.33 (d, *J* = 8.7 Hz, 1H), 7.22 (dd, *J* = 5.3, 2.4 Hz, 1H), 7.05 (app d, *J* = 8.8 Hz, 2H), 6.19 (br s, 1H); ^{13}C NMR (125 MHz, $CDCl_3$) δ 146.1, 136.1, 133.9 (2C), 129.9, 128.1, 127.8, 125.4, 120.1, 119.3, 116.7 (2C), 103.8; HRMS (ESI) m/z calcd for $C_{13}H_7Cl_2N_2$ $[M - H]^-$ 260.9992; found 260.9991.

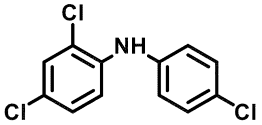

A7

(4-Chloro-phenyl)-(2,4-dichloro-phenyl)-amine (analogue A7). The method above for **A5** was employed to synthesize compound **A7** with the following stoichiometric amounts: 2,4-

dichlorobenzenamine (1.0 mmol, 0.16 g), 1-bromo-4-chlorobenzene (1.3 mmol, 0.25 g), $Pd_2(dba)_3$ [tris(dibenzylideneacetone)dipalladium(0)] (0.08 mmol, 0.07 g), BINAP (2,2′-bis(diphenylphosphino)-1,1′-binaphthyl) (0.14 mmol, 0.08 g) and sodium tert-butoxide (1.3 mmol, 0.12 g) to afford compound **A7** as brownish solid (0.16 g) in 60% yield; IR (cast film) ν_{max} = 3406, 3062, 2924, 2850, 1590, 1505, 1459, 1408, 1383, 1316, 1264, 1220, 1176, 1093, 1048, 1011, 868, 813, 759, 710, 658 cm^{-1}; ^{1}H NMR (500 MHz, $CDCl_3$) δ 7.36 (app s, 1H), 7.28 (app d, *J* = 6.7 Hz, 2H), 7.10 (d, *J* = 1.9 Hz, 2H), 7.05 (app d, *J* = 6.7 Hz, 2H), 5.99 (br s, 1H); ^{13}C NMR (125 MHz, $CDCl_3$) δ 139.8, 138.8, 129.6, 129.4 (2C), 127.9, 127.6, 124.8, 122.1, 121.6 (2C), 116.3; HRMS (EI) m/z calcd for $C_{12}H_8Cl_3N$ $[M]^+$ 270.9722; found 270.9720.

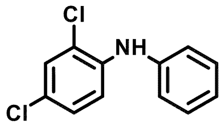

A8

2,4-Dichloro-N-phenylaniline (analogue A8). The method above for **A5** was employed to synthesize compound **A8** with the following stoichiometric amounts: 2,4-dichlorobenzenamine (1.0 mmol, 0.16 g), bromobenzene (1.3 mmol, 0.14 mL), $Pd_2(dba)_3$ [tris(dibenzylideneacetone)dipalladium(0)] (0.08 mmol, 0.07 g), BINAP (2,2′-bis(diphenylphosphino)-1,1′-binaphthyl) (0.14 mmol, 0.08 g) and sodium tert-butoxide (1.3 mmol, 0.12 g) to afford compound 9 as brownish solid (0.9 g) in 40% yield; IR (cast film) ν_{max} = 3407, 3068, 3048, 3026, 2955, 2925, 2853, 1594, 1519, 1467, 1390, 1315, 1048, 830, 807, 739, 694 cm^{-1}; ^{1}H NMR (500 MHz, $CDCl_3$) δ 7.35 (d, *J* = 2.4 Hz, 1H), 7.33 (app t, *J* = 7.9 Hz, 2H), 7.17 (d, *J* = 8.8 Hz, 1H), 7.13 (app d, *J* = 1.0 Hz, 2H), 7.09-7.05 (m, 2H), 6.04 (br s, 1H); ^{13}C NMR (125 MHz, $CDCl_3$) δ 141.1, 139.3, 129.6 (2C), 129.3, 127.6, 124.2, 123.2 (2C), 121.8, 120.5 (2C), 116.5; HRMS (EI) m/z calcd for $C_{12}H_9Cl_2N$ $[M]^+$ 237.0112 ; found 237.0111.

References.

1. M. E. Lanning, W. Yu, J. L. Yap, J. Chauhan, L. Chen, E. Whiting, L. S. Pidugu, T. Atkinson, H. Bailey, W. Li, B. M. Roth, L. Hynicka, K. Chesko, E. A. Toth, P. Shapiro, A. D. MacKerell, Jr., P. T. Wilder, and S. Fletcher. "Structure-based design of N-substituted 1-hydroxy-4-sulfamoyl-2-naphthoates as selective inhibitors of the Mcl-1 oncoprotein." *Eur. J. Med. Chem. 113* (**2016**): 273-292.

2. S. Kathiravan and I. A. Nicholls. "Monoprotected l-Amino Acid (l-MPAA), Accelerated Bromination, Chlorination, and Iodination of C (sp2)− H Bonds by Iridium (III) Catalysis." *Chem.-Eu.r J, 23,* (**2017**): 7031-7036.
3. X.-L. Zhu, R. Zhang, Q.-Y. Wu, Y.-J. Song, Y.-X. Wang, J.-F. Yang, and G.-F. Yang. "Natural product neopeltolide as a cytochrome bc_1 complex inhibitor: mechanism of action and structural modification." *J. Agric. Food Chem. 67,* (**2019**): 2774-2781.
4. J. P. Wolfe, S. Wagaw, and S. L. Buchwald. "An improved catalyst system for aromatic carbon− nitrogen bond formation: the possible involvement of bis (phosphine) palladium complexes as key intermediates." *J. Am. Chem. Soc. 118,* (**1996**): 7215-7216.

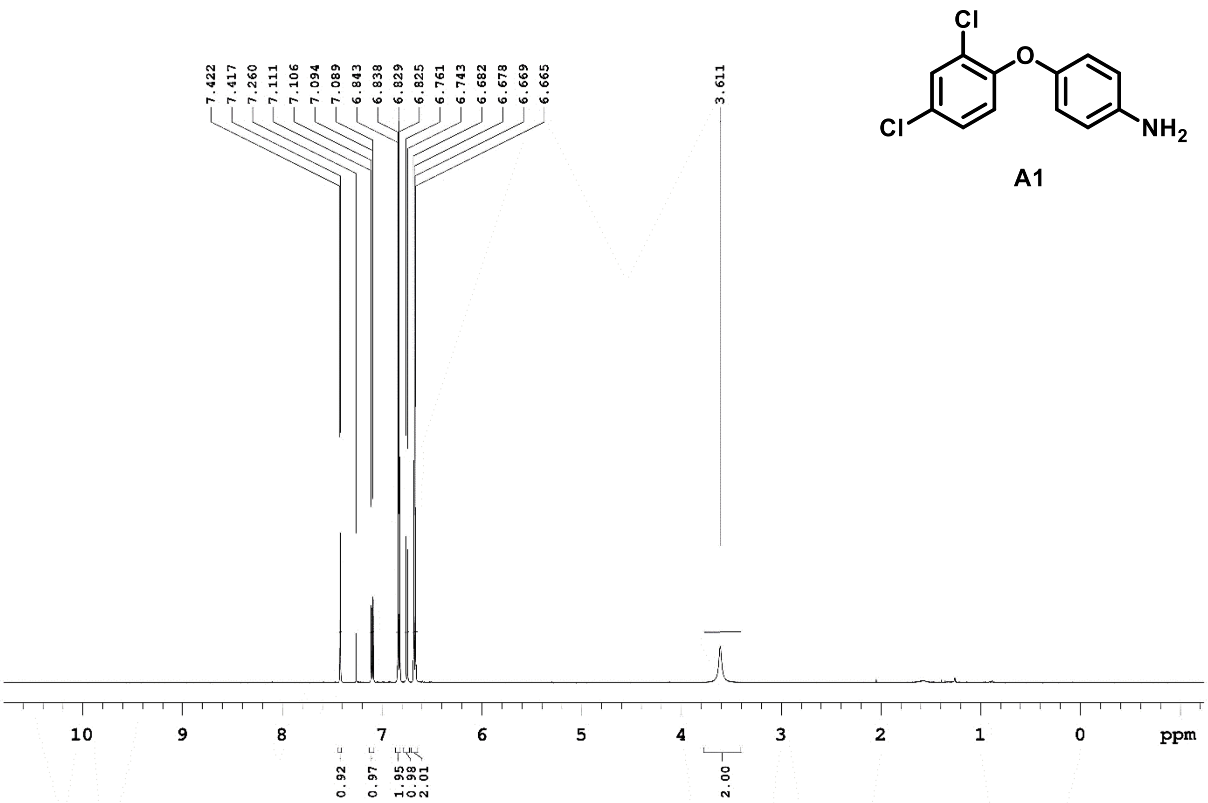
7.422
7.417
7.260
7.111
7.106
7.094
7.089
6.843
6.838
6.829
6.825
6.761
6.743
6.682
6.678
6.669
6.665
3.611
Cl
O
Cl
NH2
A1
10
9
8
7
6
5
4
3
2
1
0
ppm
0.92
0.97
1.95
0.98
2.01
2.00

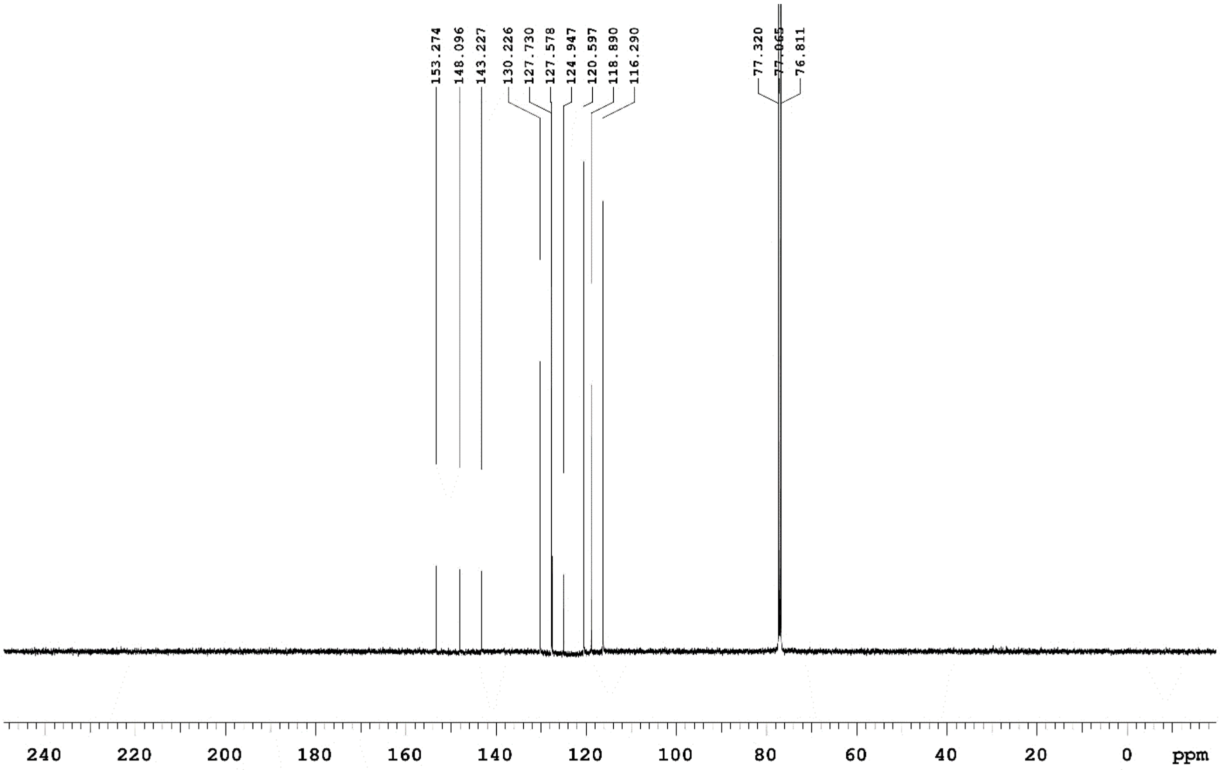
153.274
148.096
143.227
130.226
127.730
127.578
124.947
120.597
118.890
116.290
77.320
77.065
76.811
240
220
200
180
160
140
120
100
80
60
40
20
0
ppm

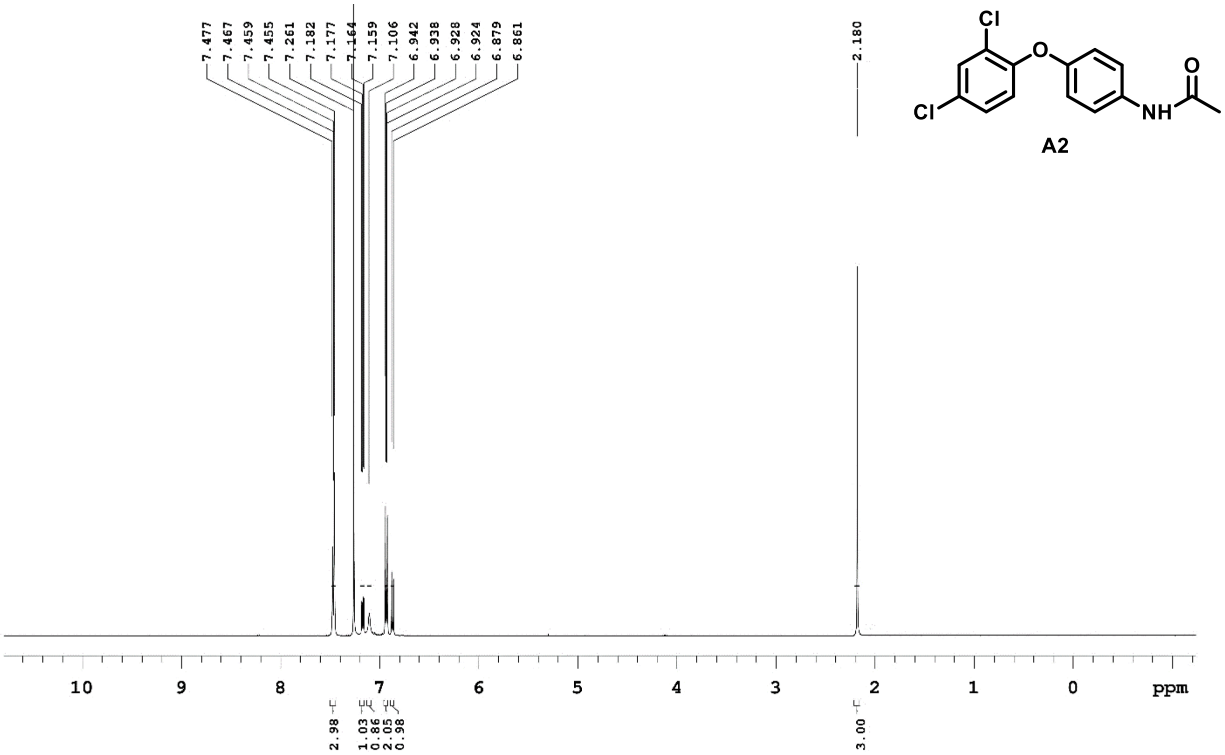

A2

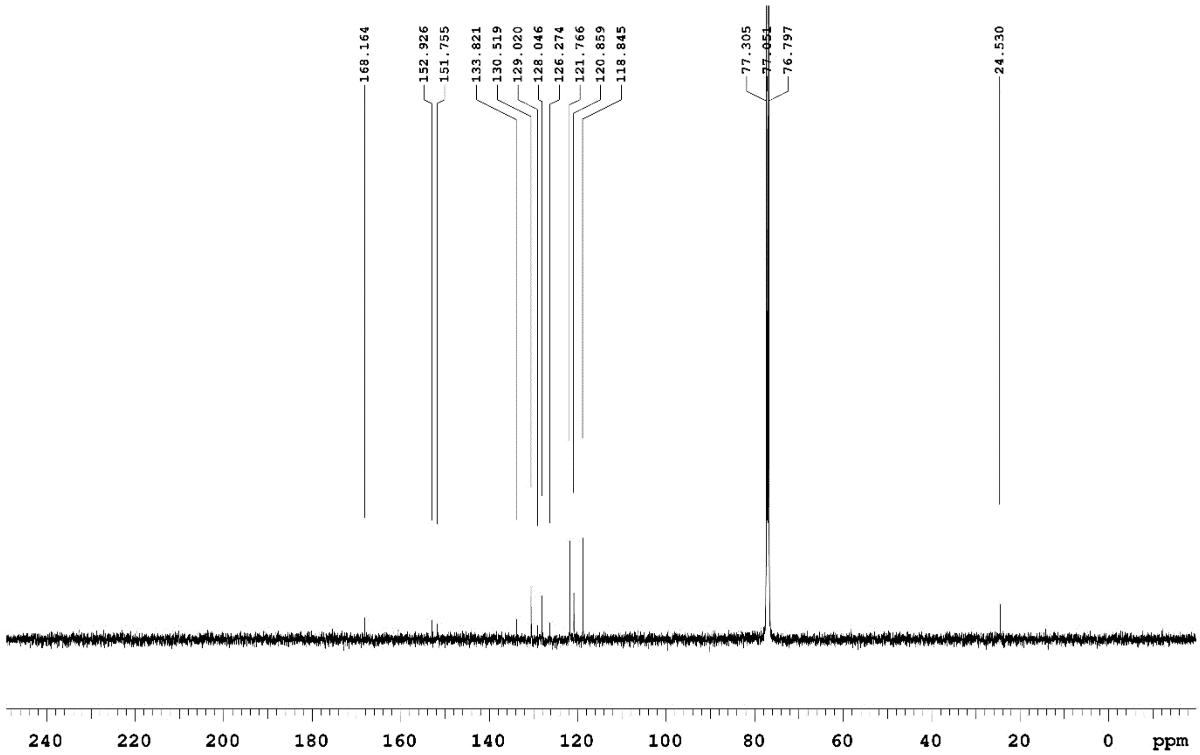

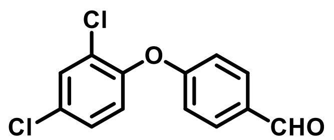
Cl
O
Cl
CHO
A3

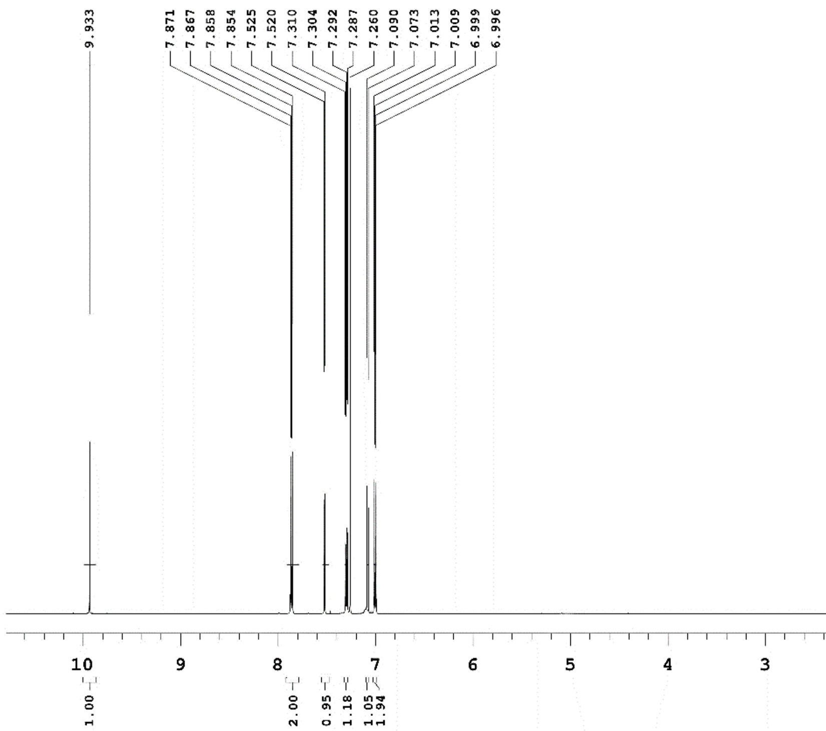
9.933
7.871
7.867
7.858
7.854
7.525
7.520
7.310
7.304
7.292
7.287
7.260
7.090
7.073
7.013
7.009
6.999
6.996
10
9
8
7
6
5
4
3
2
1
0
ppm
1.00
2.00
0.95
1.18
1.05
1.94

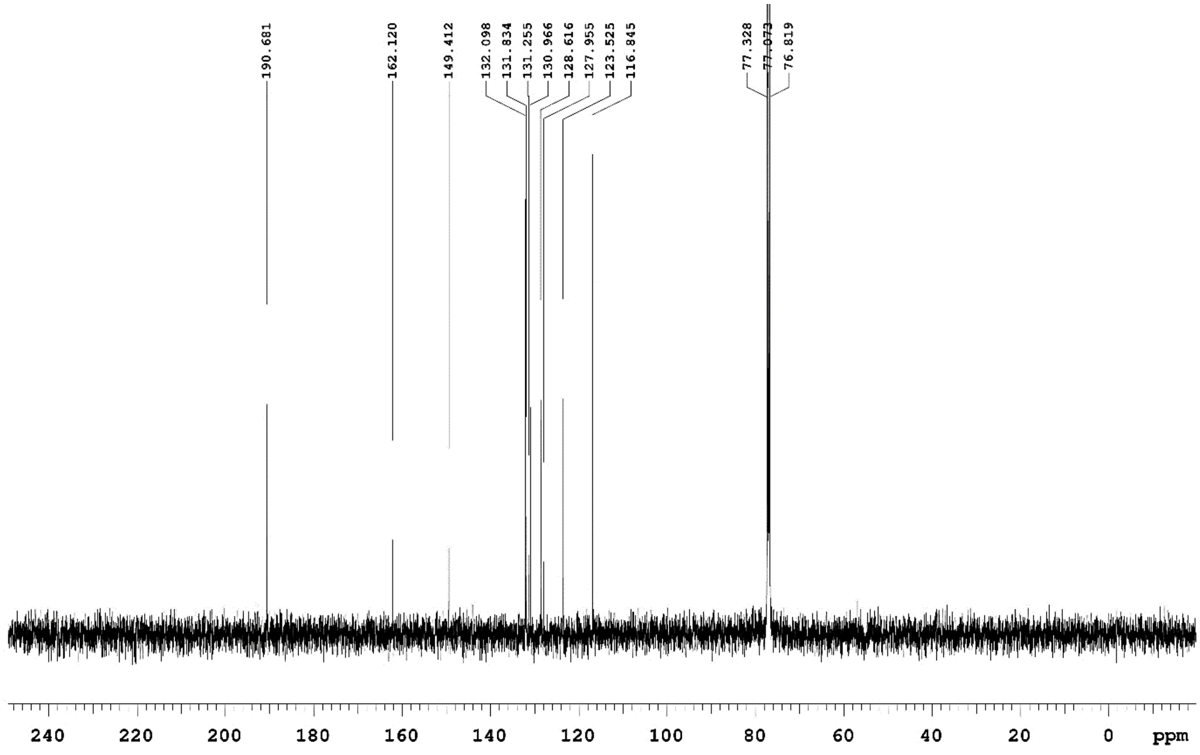
190.681
162.120
149.412
132.098
131.834
131.255
130.966
128.616
127.955
123.525
116.845
77.328
77.073
76.819
240
220
200
180
160
140
120
100
80
60
40
20
0
ppm

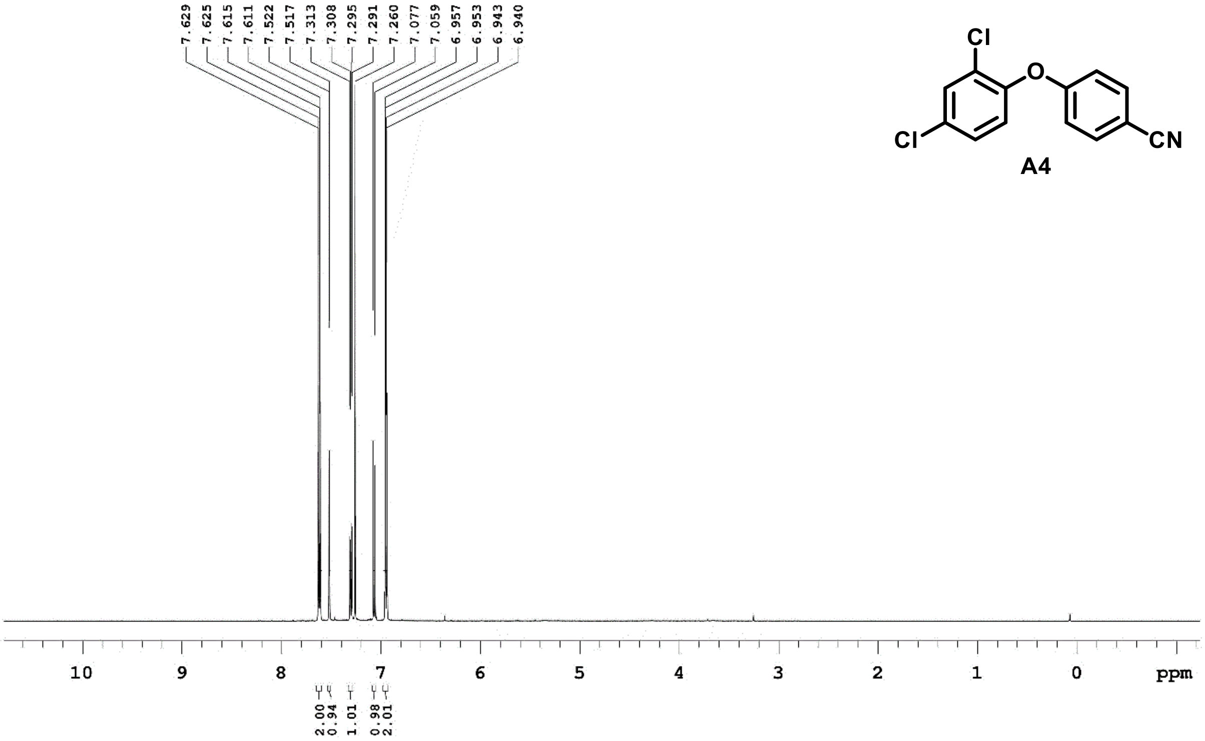
Cl
O
Cl
CN
A4
7.629
7.625
7.615
7.611
7.522
7.517
7.313
7.308
7.295
7.291
7.260
7.077
7.059
6.957
6.953
6.943
6.940
10
9
8
7
6
5
4
3
2
1
0
ppm
2.00
0.94
1.01
0.98
2.01

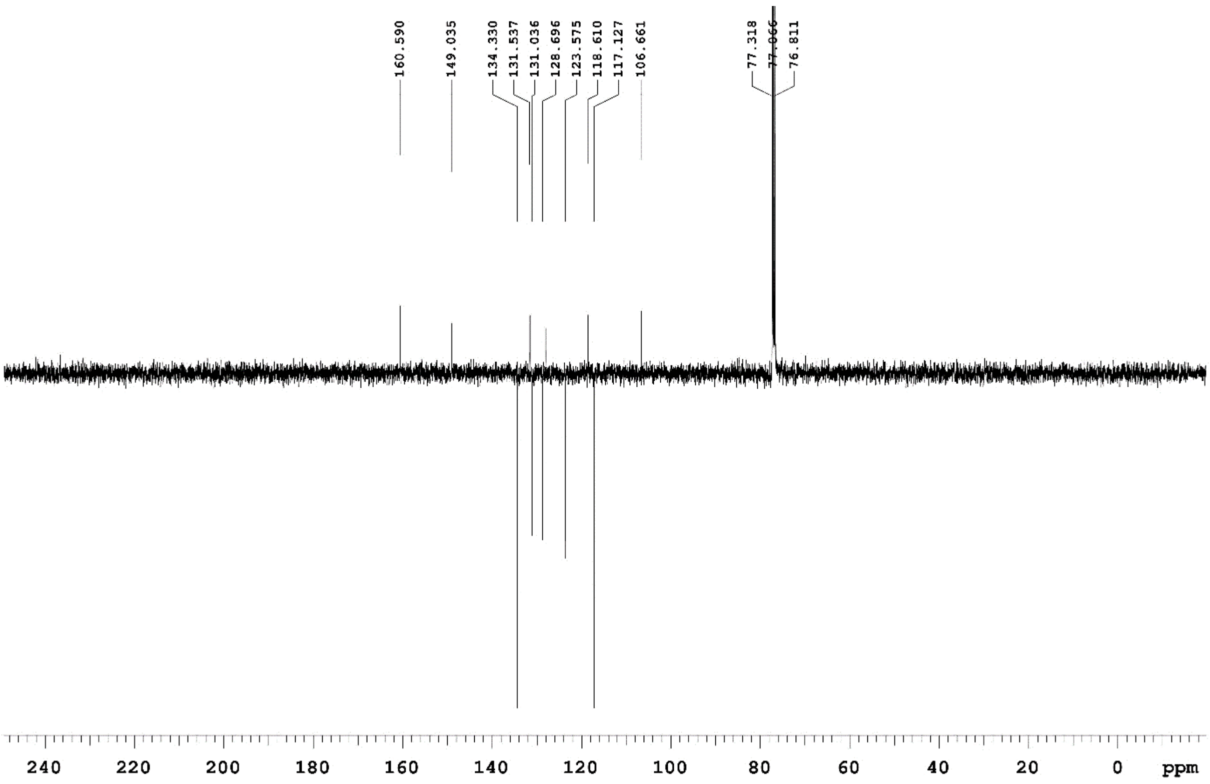
160.590
149.035
134.330
131.537
131.036
128.696
123.575
118.610
117.127
106.661
77.318
77.066
76.811
240
220
200
180
160
140
120
100
80
60
40
20
0
ppm

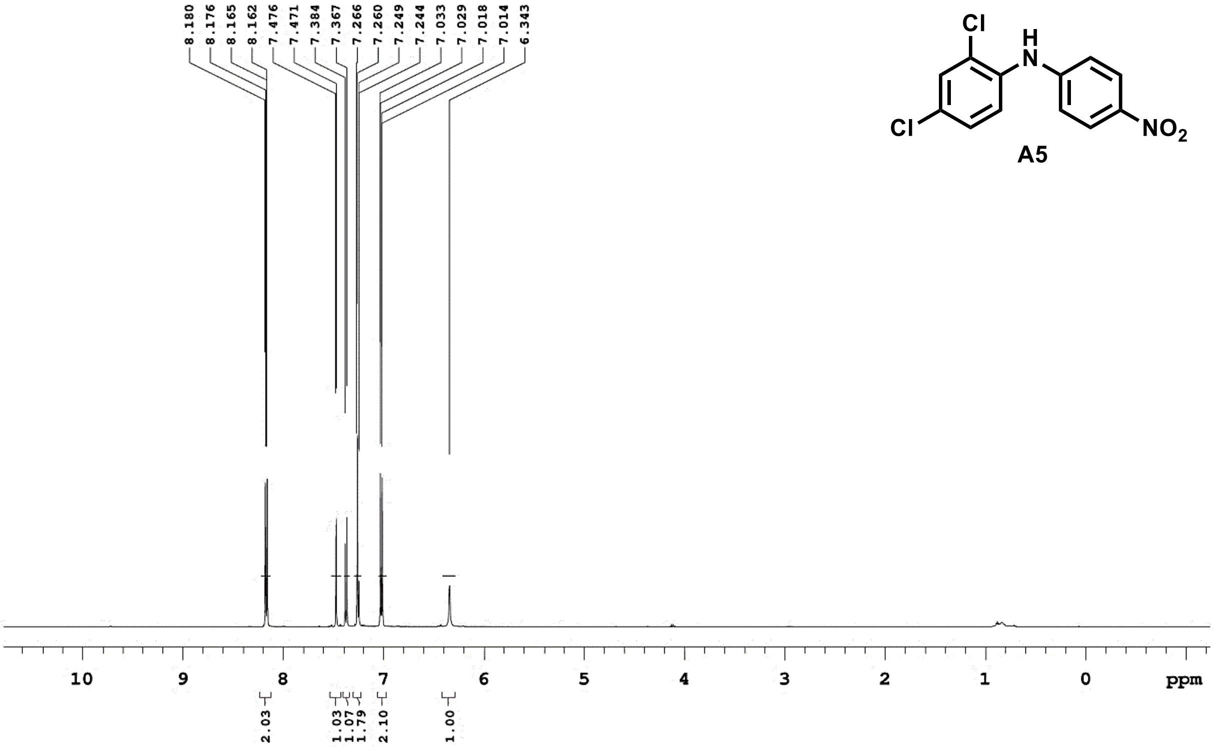

Cl
H
N
Cl
NO2
A5
8.180
8.176
8.165
8.162
7.476
7.471
7.384
7.367
7.266
7.260
7.249
7.244
7.033
7.029
7.018
7.014
6.343
10
9
8
7
6
5
4
3
2
1
0
ppm
2.03
1.03
1.07
1.79
2.10
1.00

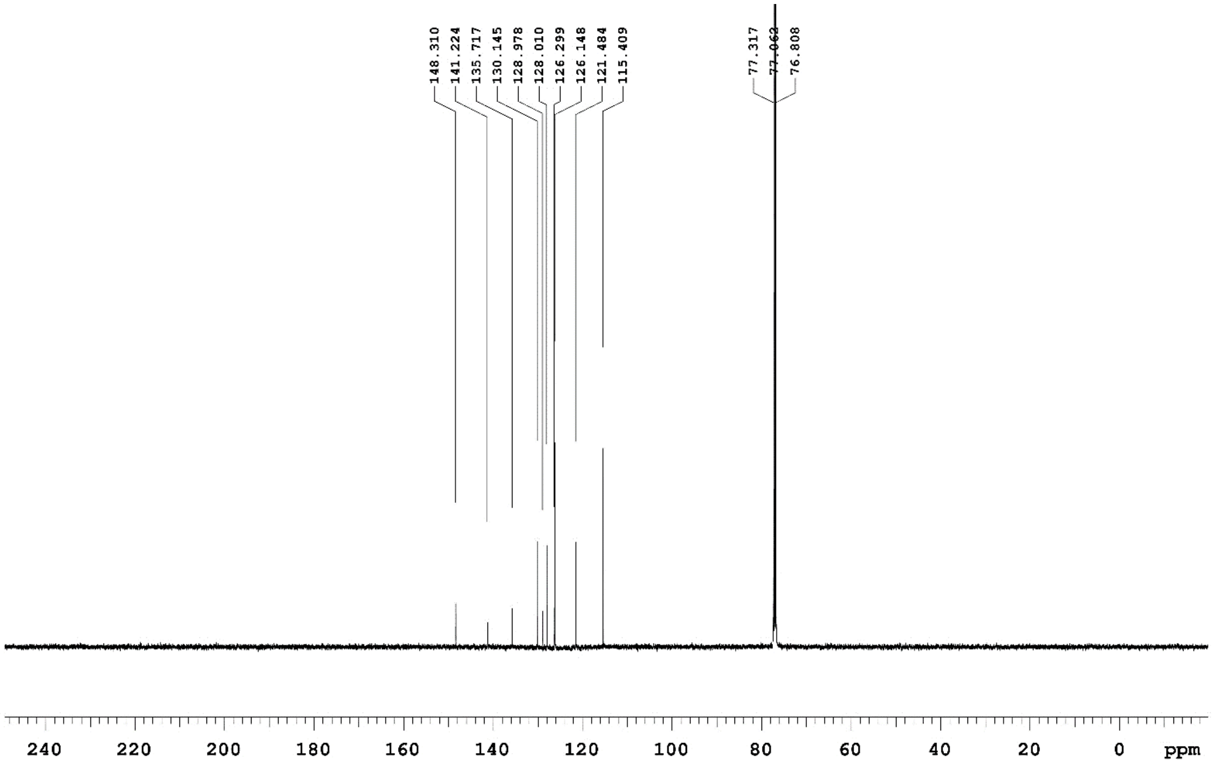

148.310
141.224
135.717
130.145
128.978
128.010
126.299
126.148
121.484
115.409
77.317
77.062
76.808
240
220
200
180
160
140
120
100
80
60
40
20
0
ppm

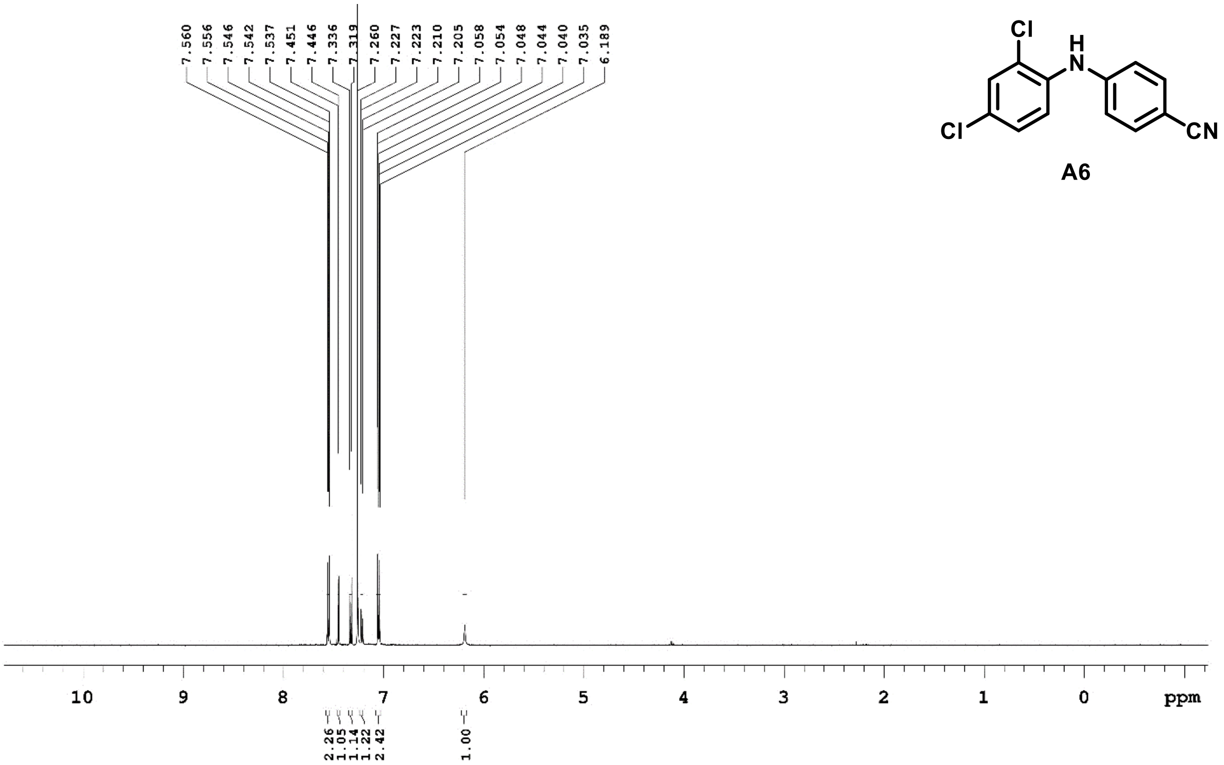

7.560 7.556 7.546 7.542 7.537 7.451 7.446 7.336 7.319 7.260 7.227 7.223 7.210 7.205 7.058 7.054 7.048 7.044 7.040 7.035 6.189
10 9 8 7 6 5 4 3 2 1 0 ppm
2.26 1.05 1.14 1.22 2.42 1.00
Cl
H
N
Cl
CN

A6

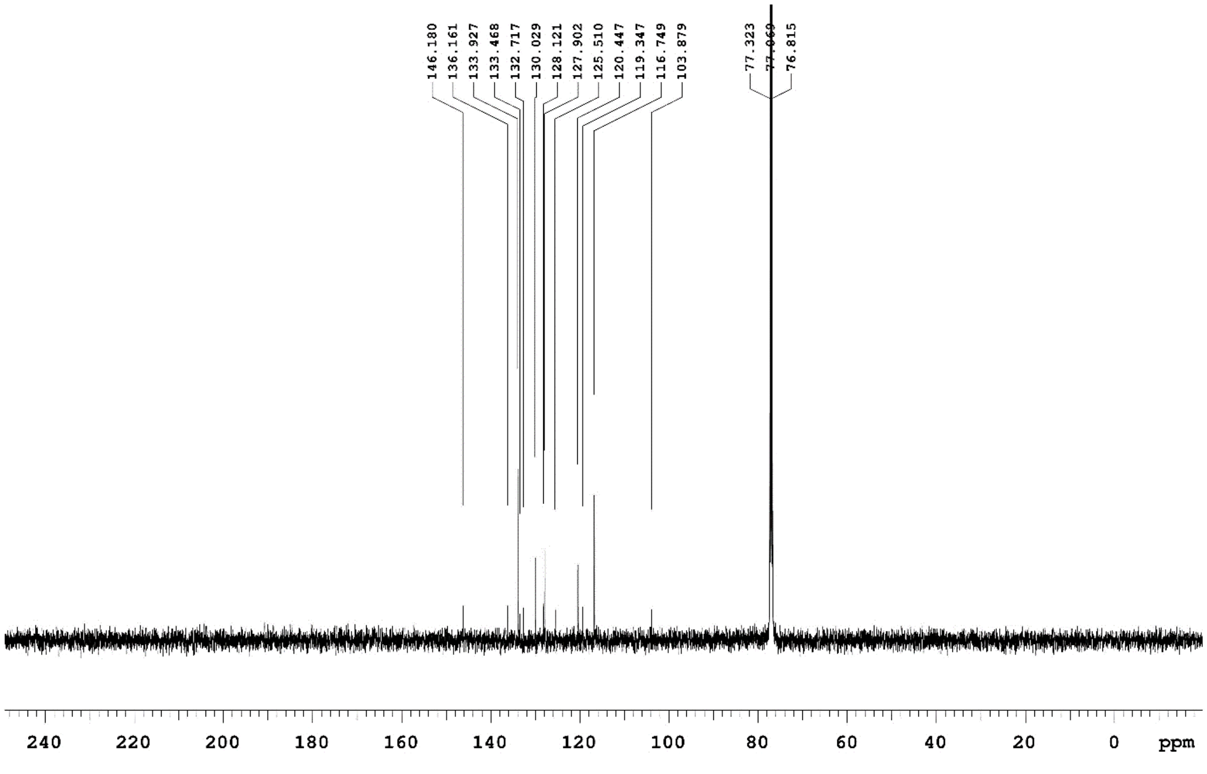

146.180 136.161 133.927 133.468 132.717 130.029 128.121 127.902 125.510 120.447 119.347 116.749 103.879
77.323 77.069 76.815
240 220 200 180 160 140 120 100 80 60 40 20 0 ppm

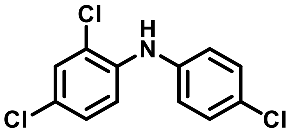
Cl
H
N
Cl
Cl

A7

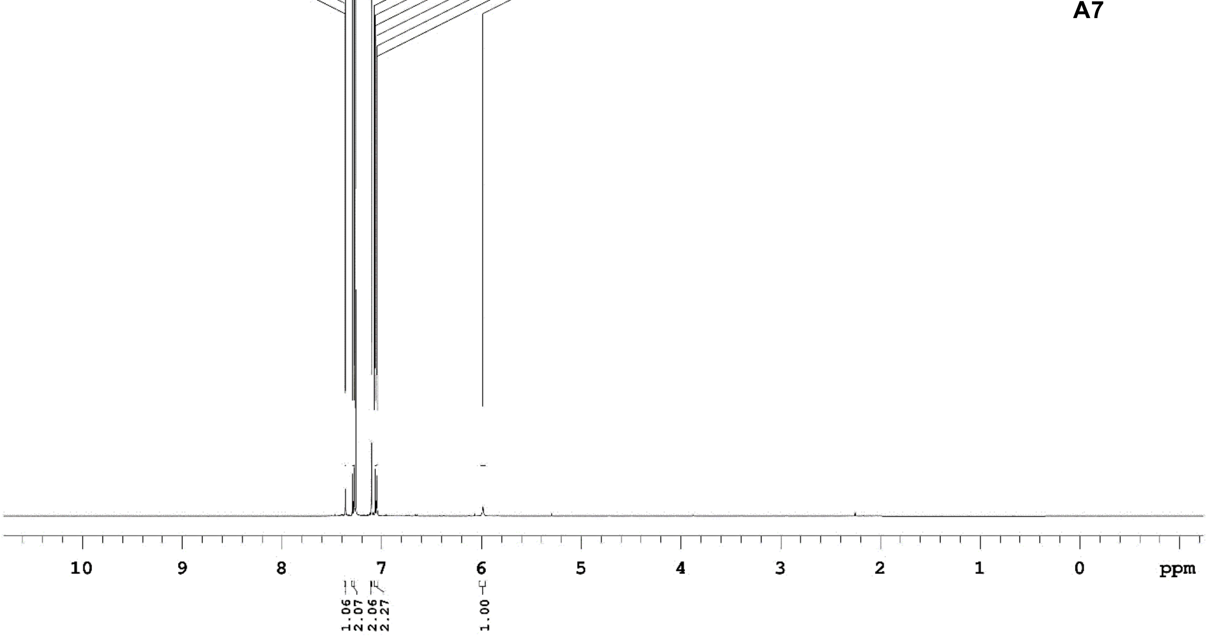
7.367
7.365
7.364
7.362
7.292
7.288
7.279
7.274
7.268
7.260
7.104
7.100
7.070
7.064
7.060
7.051
7.047
7.040
5.987
10 9 8 7 6 5 4 3 2 1 0 ppm
1.06
2.07
2.06
2.27
1.00

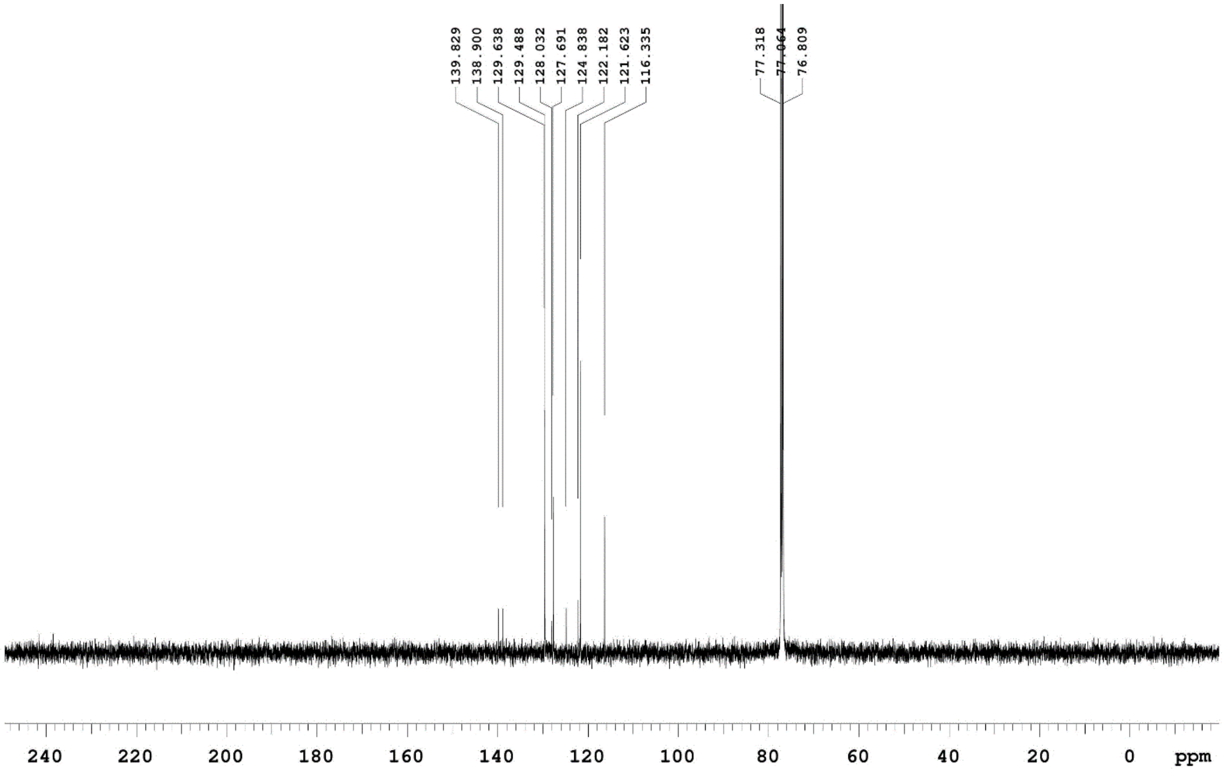
139.829
138.900
129.638
129.488
128.032
127.691
124.838
122.182
121.623
116.335
77.318
77.064
76.809
240 220 200 180 160 140 120 100 80 60 40 20 0 ppm

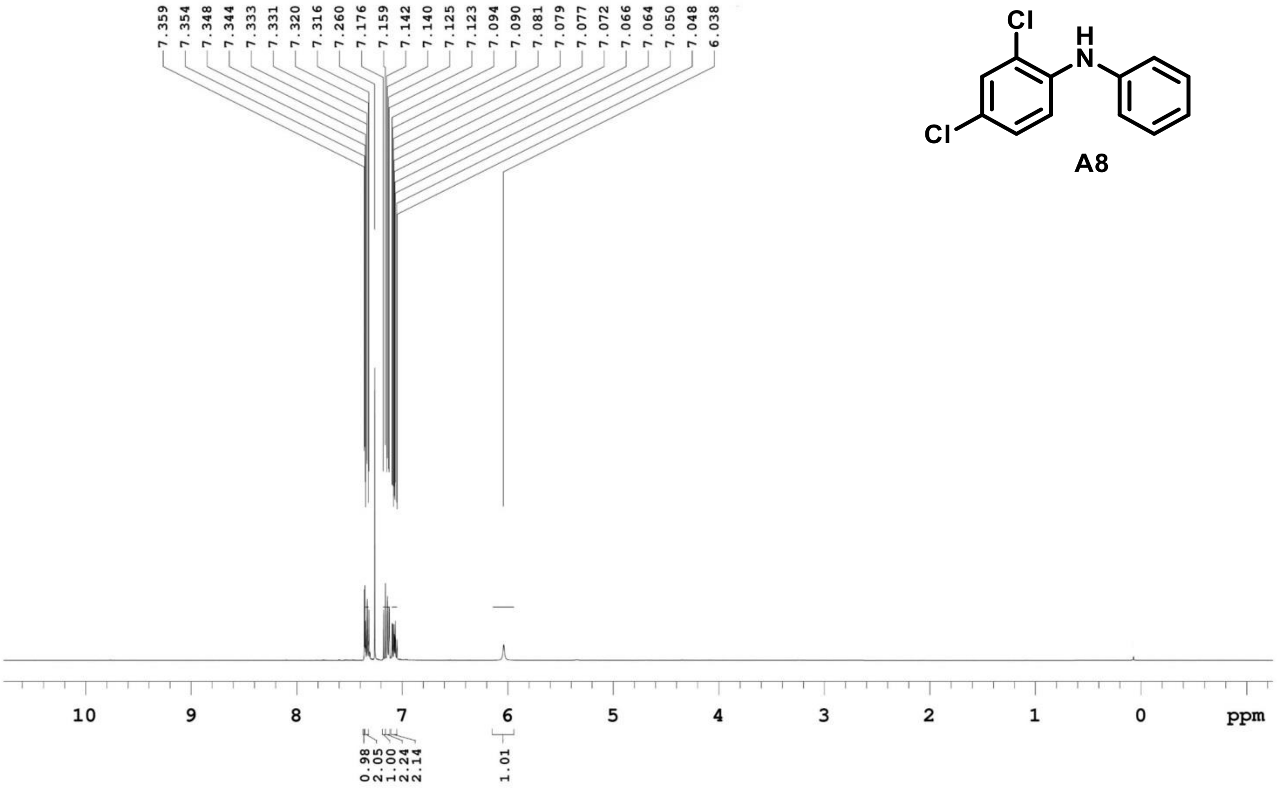

A8

7.359
7.354
7.348
7.344
7.333
7.331
7.320
7.316
7.260
7.176
7.159
7.142
7.140
7.125
7.123
7.094
7.090
7.081
7.079
7.077
7.072
7.066
7.064
7.050
7.048
6.038
10
9
8
7
6
5
4
3
2
1
0
ppm
0.98
2.05
1.00
2.24
2.14
1.01

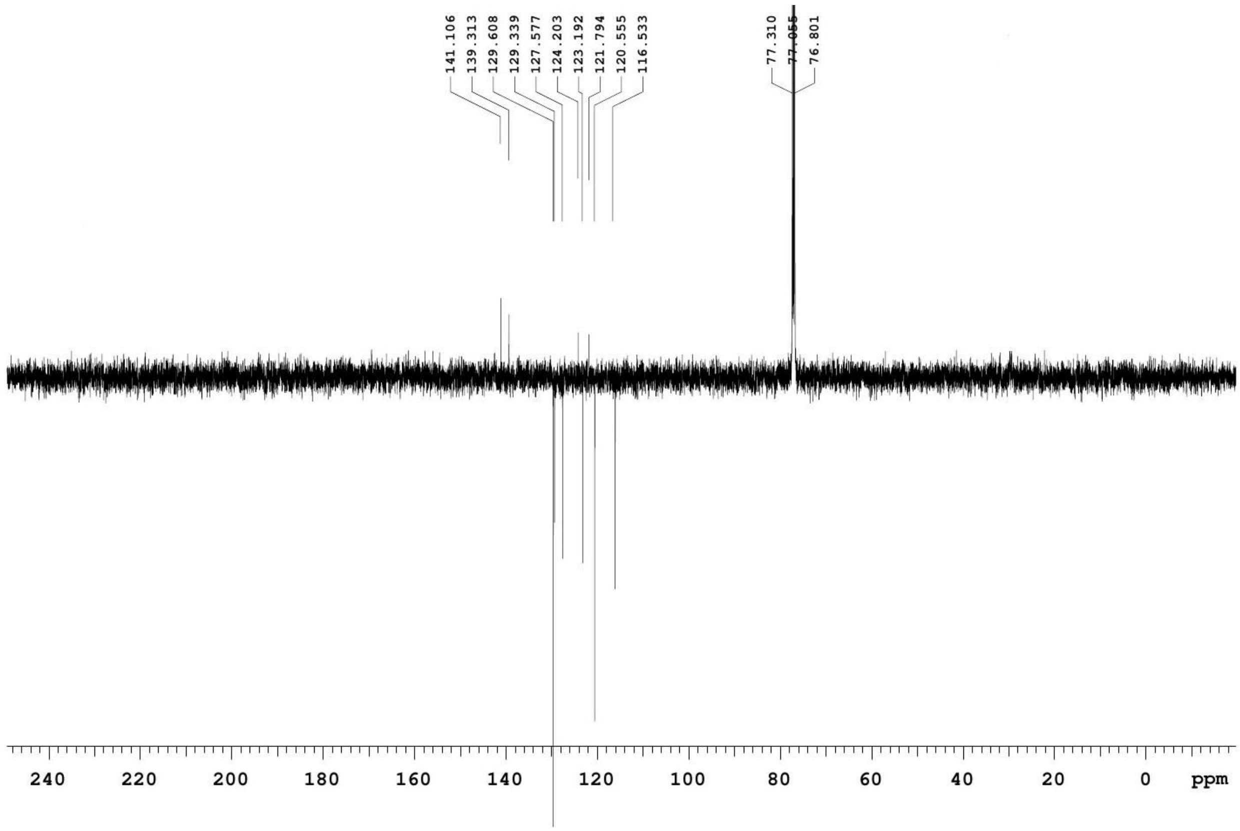
141.106
139.313
129.608
129.339
127.577
124.203
123.192
121.794
120.555
116.533
77.310
77.055
76.801
240
220
200
180
160
140
120
100
80
60
40
20
0
ppm

Inhibition of triple negative breast cancer metastasis/invasiveness by novel drugs that target epithelial to mesenchymal transition

Elizabeth Garcia ξ#, Ismat Lunaψ#, Kaya L Persadξ, Kate Agopsowiczʖ, David A Jayξ, Frederick G Westψ, Mary M Hittʖ, Sujata Persadξ*

Supplementary Figures

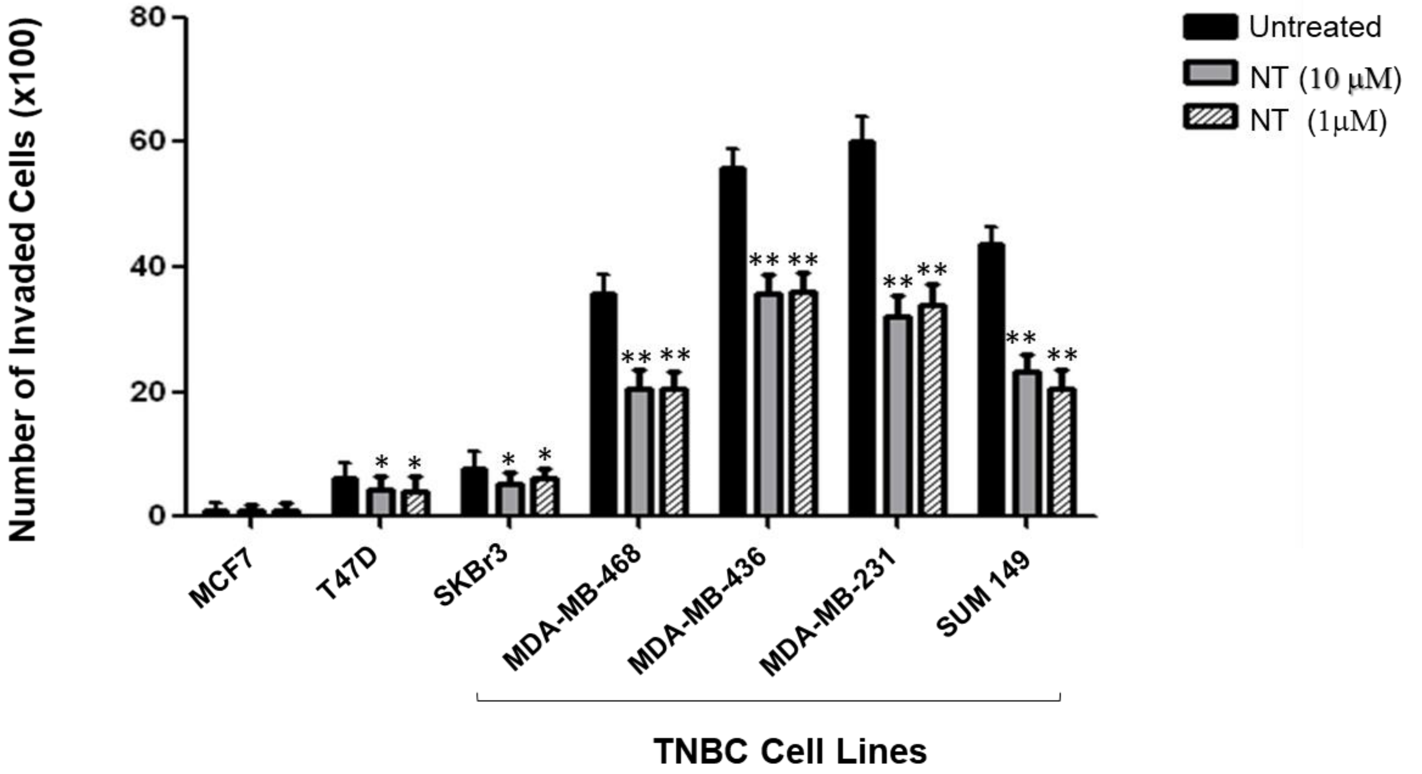

Figure S1. Matrigel invasion assay of breast cancer cell lines with and without nitrofen treatment (1µM and 10µM). TNBC cell lines (MDA-MB-468, MDA-MB-436, MDA-MB-231, MDA-MB-231-Luc, SUM 149) showed higher invasive potential than non-TNBC cell lines (MCF7, T47D, SKbr3). Nitrofen treatment (1 & 10 µM) reduced invasive potential of TNBC lines to a greater extent than non-TNBC lines. Data representative of 4 separate experiments. *p<0.05 vs. untreated; ** p<0.01 vs. untreated. [NT= Nitrofen]

Garcia et al. Figure S1

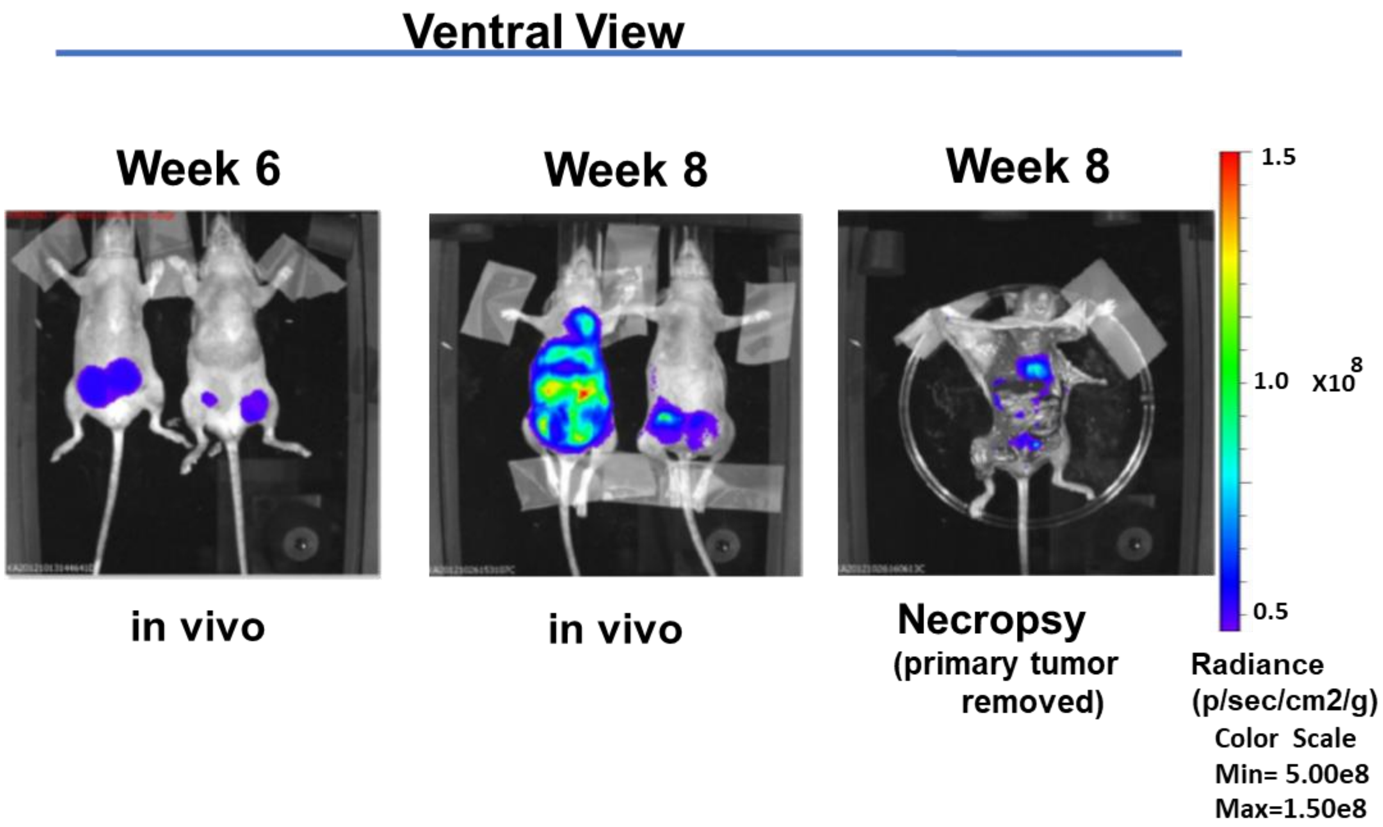

Figure S2. Growth of untreated 231-Luc tumors in the mammary fat pad. Representative bioluminescence image of human MDA-MB-231-Luc-D3H2LN (Caliper Life Sciences) breast tumors established in bilateral abdominal mammary fat pads of NIH-III nude mice. Images were acquired following s.c. injection with 15mg/kg luciferin. By week 8, metastases were consistently and clearly visible in tumor-bearing mice by in vivo and/or ex vivo imaging.

Garcia et al. Figure S2

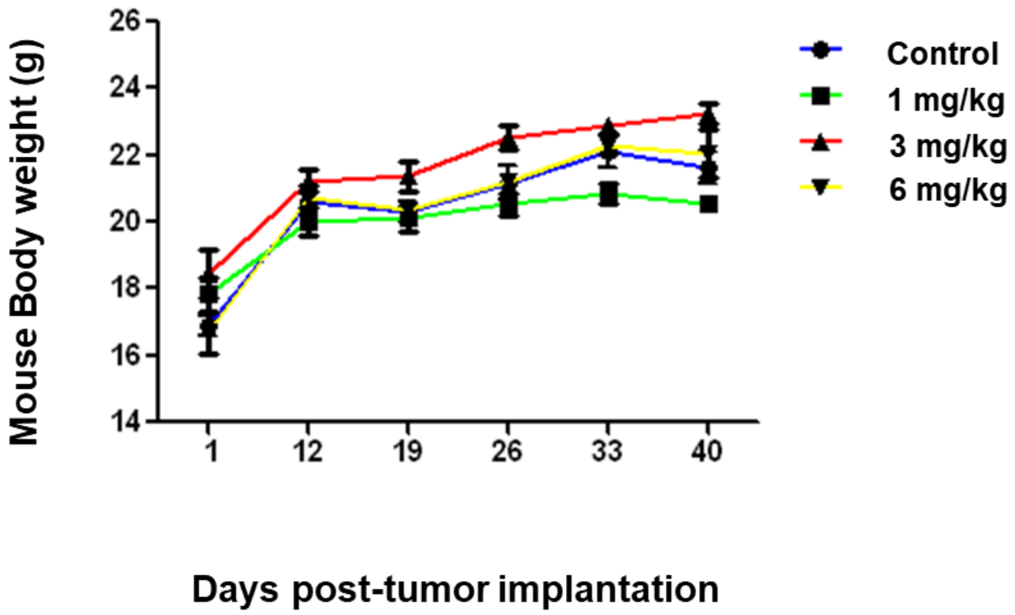

Figure S3. Body weight of mice under various treatment conditions. Body weight of mice was monitored over the duration of the various treatment conditions. There was no significant difference in the body weight of mice between the various treatment conditions. n=5-10 tumor bearing mice per group.

Garcia et al. Figure S3

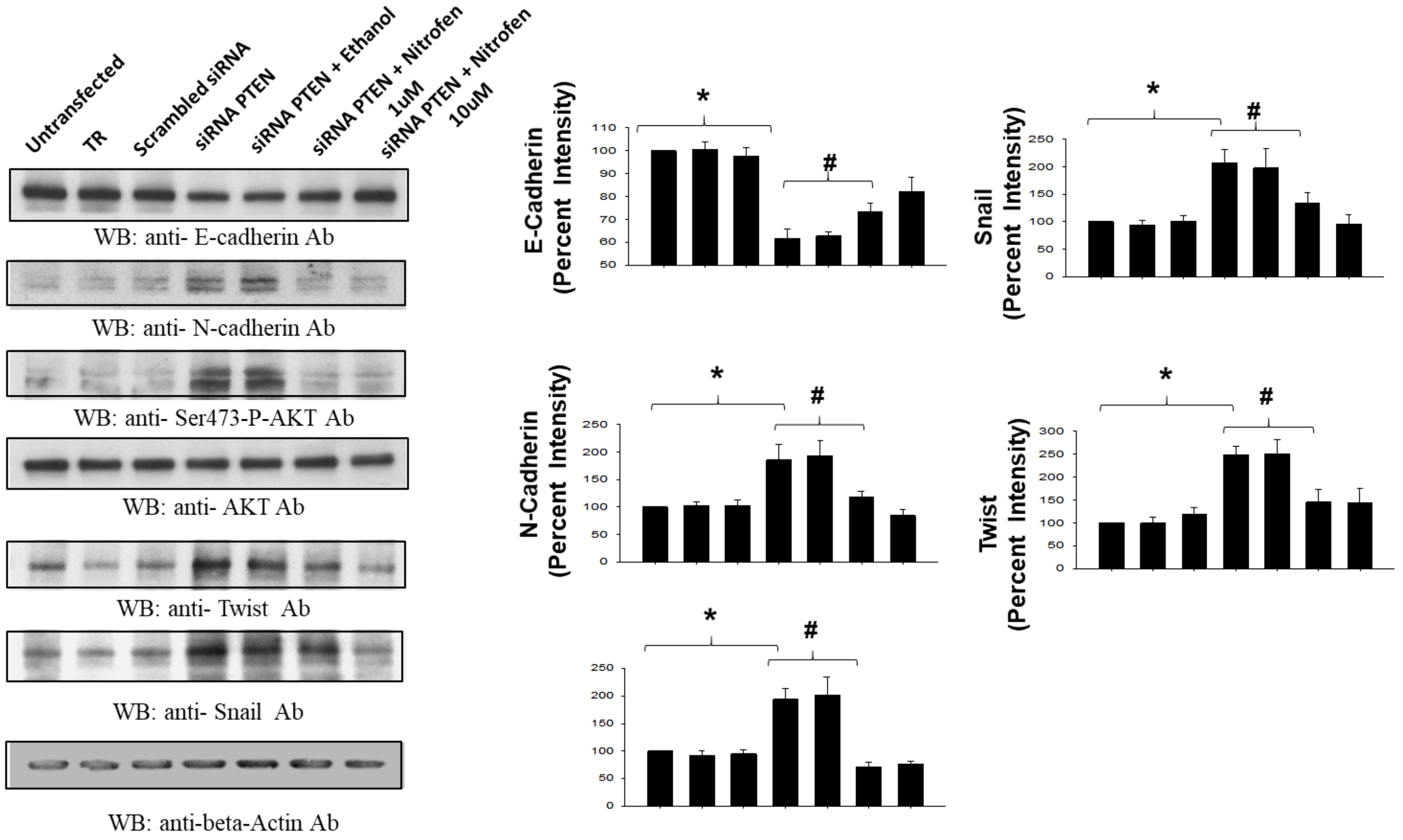

Figure S4. Nitrofen promotes MET changes in a PTEN-knockdown model of EMT of DU145 prostate cancer cells. Knockdown of PTEN resulted in down-regulation of E-cadherin, up-regulation of N-cadherin and increase in the protein levels of the transcription factors Snail and Twist. These alterations were significantly attenuated in the presence of 1 µM and 10 µM nitrofen for 24 hours. Data representative of 6 separate experiments each done in triplicate. *significantly altered from untransfected control cells p<0.01; # significantly altered from siRNA-PTEN transfected cells p<0.01. [TR=Transfection reagent].

Garcia et al. Figure S4

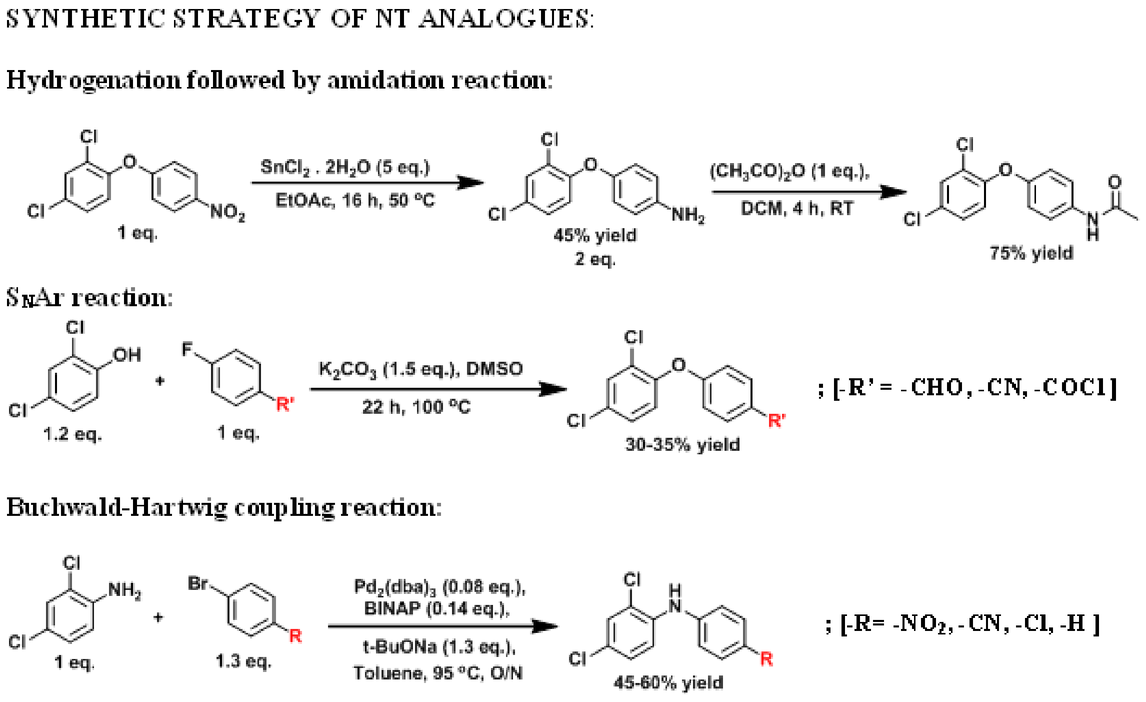

Figure S5. Synthetic routes to analogues A1-A8: reduction/acylation of nitro group, formation of diaryl ether via nucleophilic aromatic substitution, or palladium-catalyzed aromatic amination via Buchwald-Hartwig coupling reaction.

Garcia et al. Figure S5

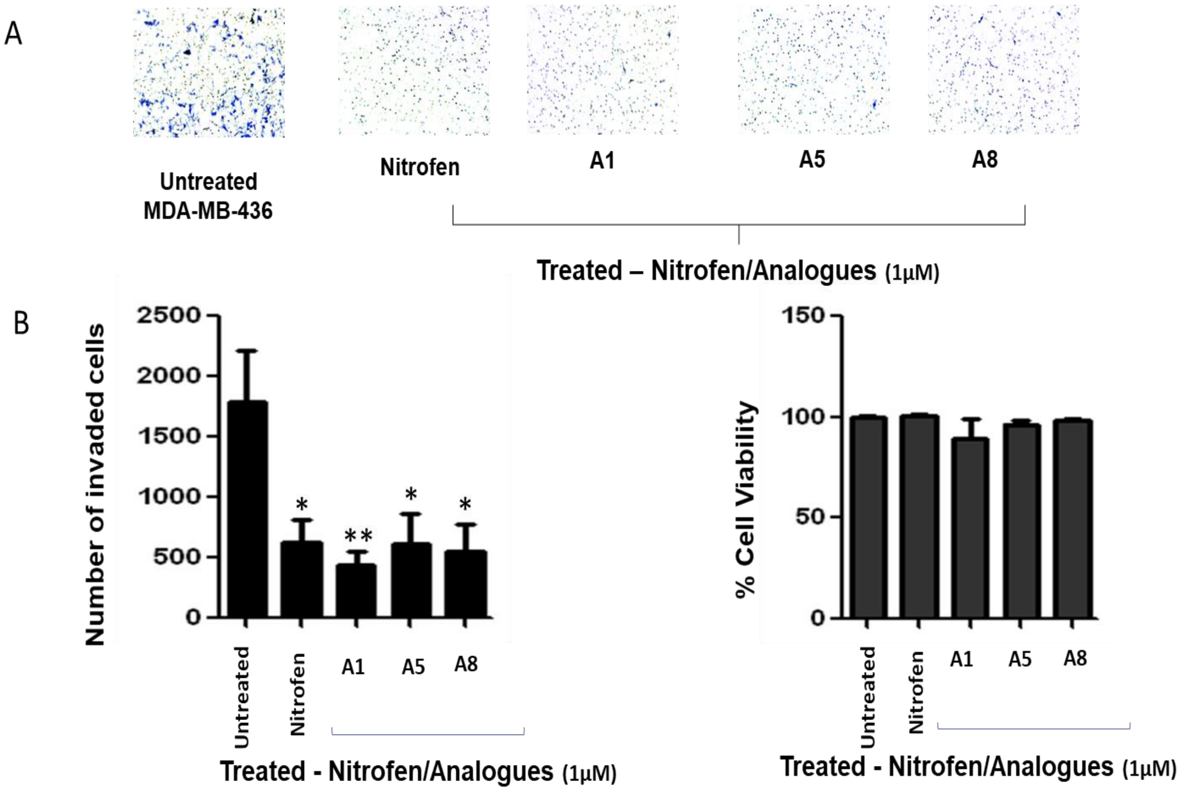

Figure S6. Photomicrograph of invading MDA-MB-436 TNBC cells in the presence or absence of treatment with nitrofen and analogues A1, A5 and A8. B. Quantification of Matrigel invasion assay of MDA-MB-436 cells treated with nitrofen and nitrofen analogues A1, A5 and A8. Data show that analogues A1, A5 and A8 significantly reduce the invasive potential of MDA-MB-436 cells. C. AlamarBlue cell viability assay done under the same conditions shows that there is no change in relative viability of cells with treatments. Data representative of 6 separate experiments each done in triplicate. *significantly altered from untreated control $p<0.05$; ** significantly altered from untreated control $p<0.01$.

Garcia et al. Figure S6

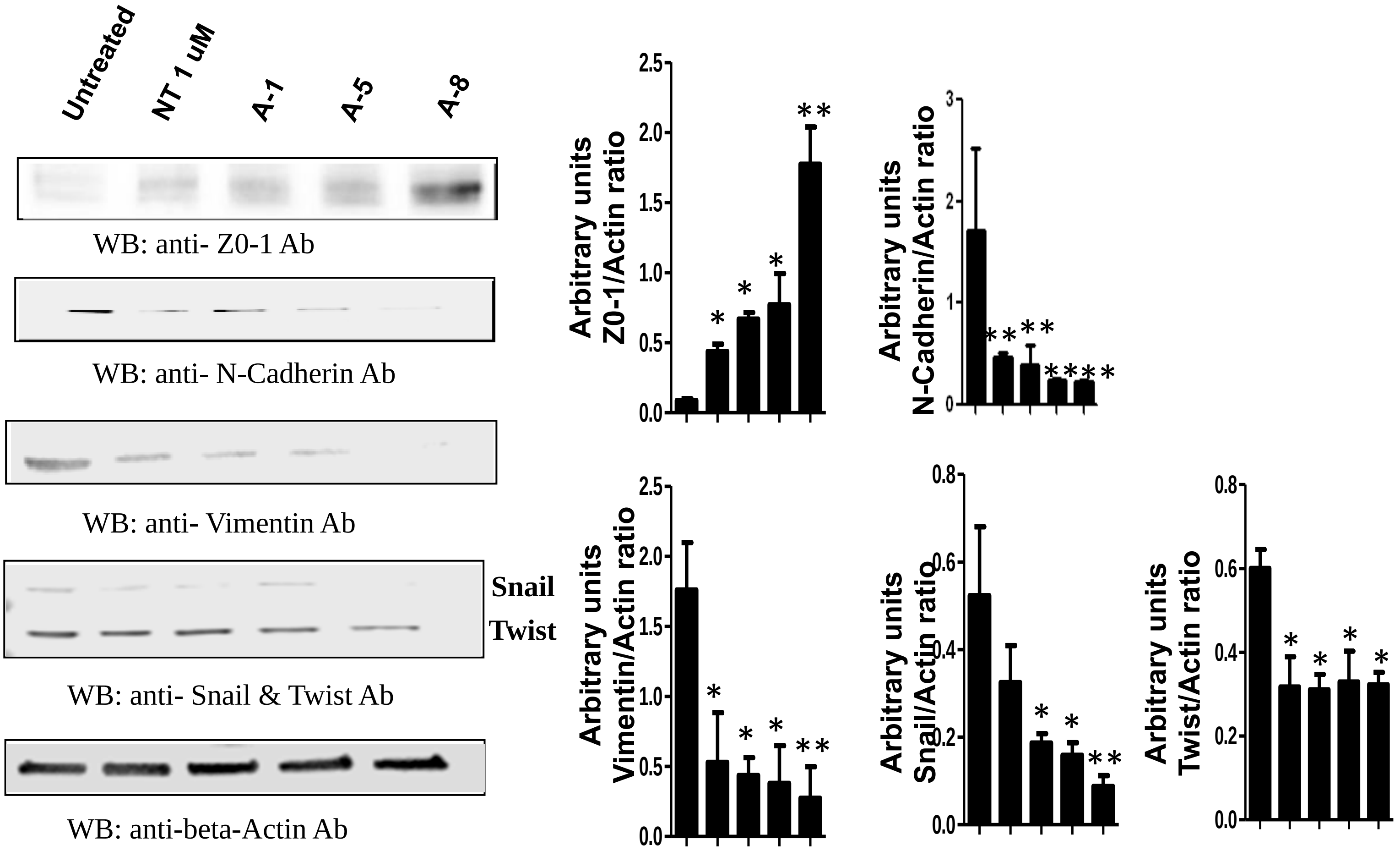

Figure S7. Nitrofen analogues promote mesenchymal to epithelial transformation in MDA-MB-436 TNBC cell line. Western blot analysis shows that treatment with nitrofen and analogues A1, A5 and A8 at 1µM concentration for 24 hours results in the appearance of epithelial marker ZO-1 in MDA-MB-436 cell line which is mesenchymal-like in phenotype. Treatment with nitrofen and analogues A1, A5 and A8 results in significant decrease in cellular expression of mesenchymal markers N-cadherin, vimentin, twist and snail in MDA-MB-436 cell line. Data representative of 6 separate experiments each done in triplicate. *significantly altered from untreated control p<0.05; **significantly altered from untreated control p<0.01.

Garcia et al. Figure S7

Inhibition of triple negative breast cancer metastasis/invasiveness by novel drugs that target epithelial to mesenchymal transition.

Elizabeth Garcia [§#], Ismat Luna[ψ#], Kaya L Persad[§], Kate Agopsowicz[ʅ], David A Jay[§], Frederick G West[ψ], Mary M Hitt[ʅ], Sujata Persad[§*]

Supplementary Information File

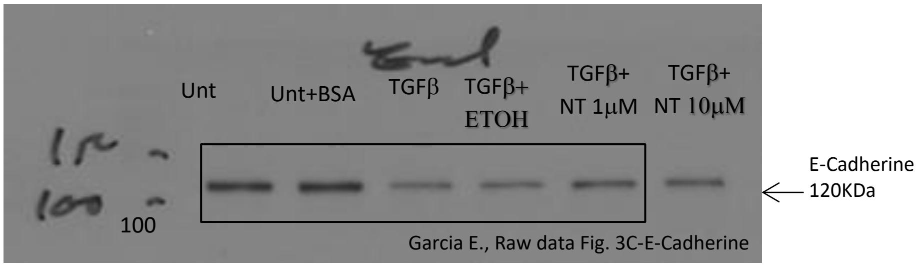

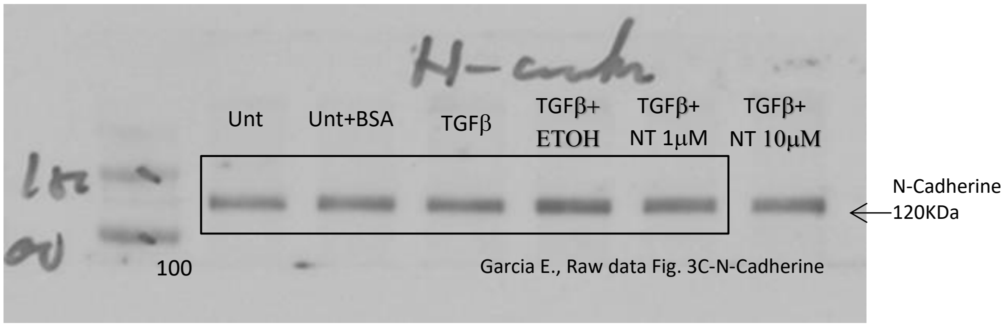

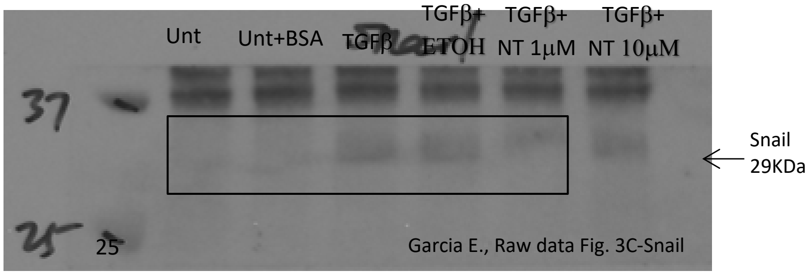

Garcia E., Raw data Fig. 3C

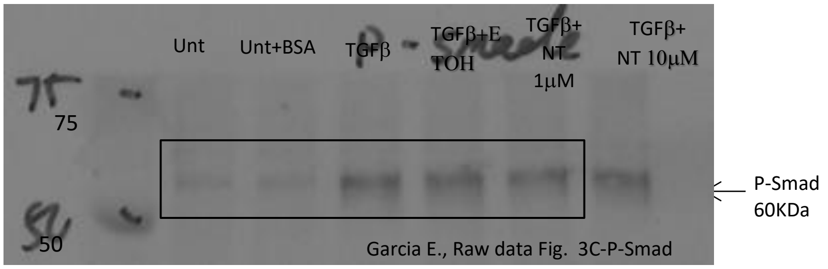

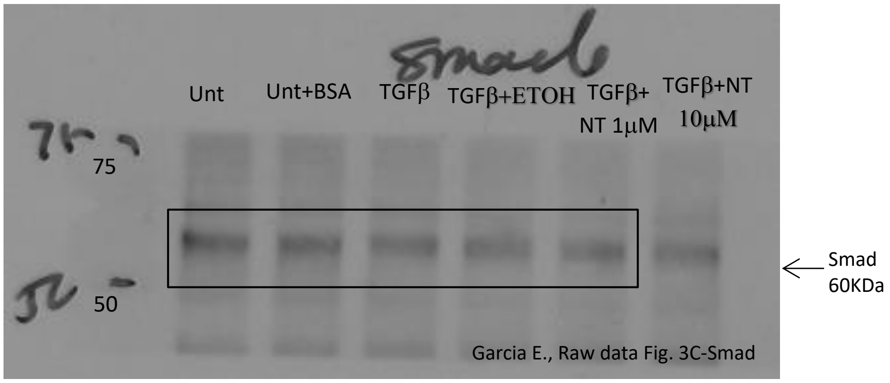

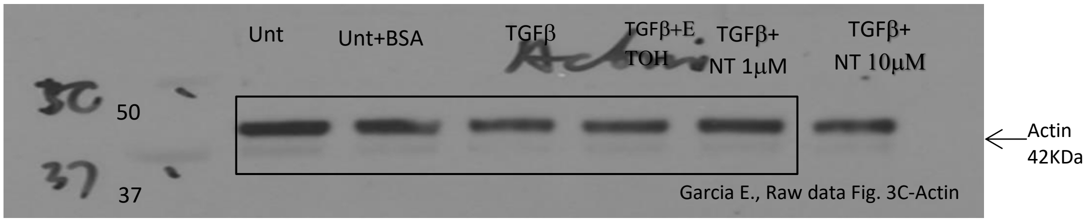

Garcia E., Raw data Fig. 3C

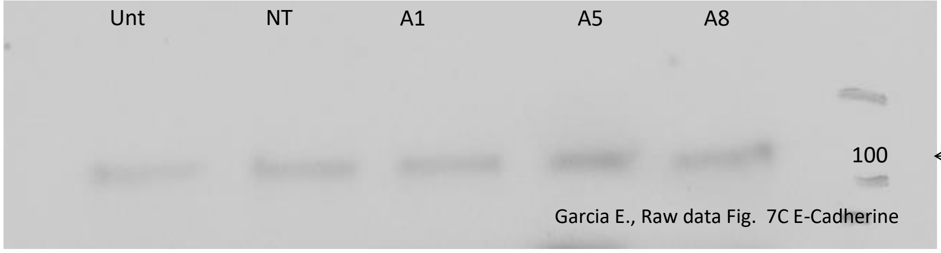

E-Cadherine
120KDa

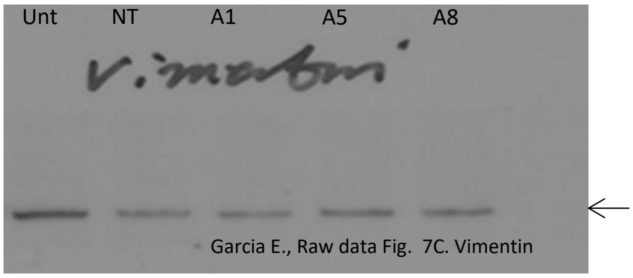

Vimentin
57KDa

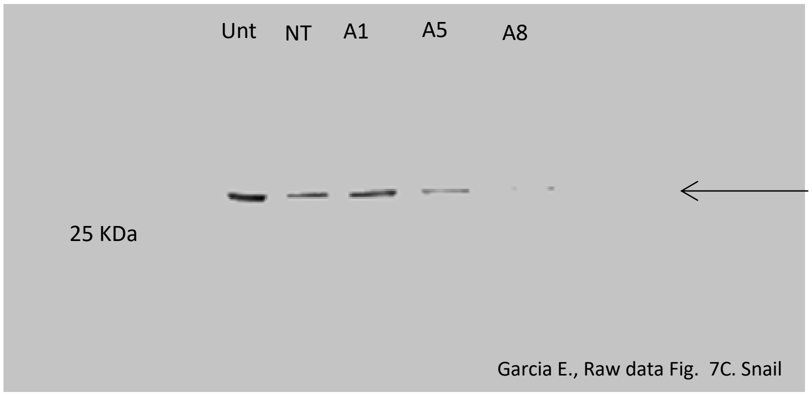

Snail
29KDa

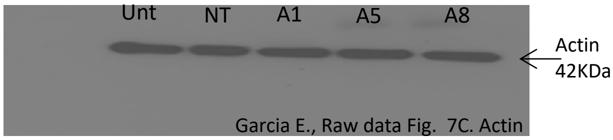

Actin
42KDa

Garcia E., Raw data Fig. 7C

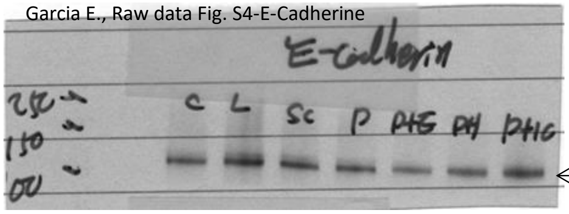

E-Cadherine 120KDa

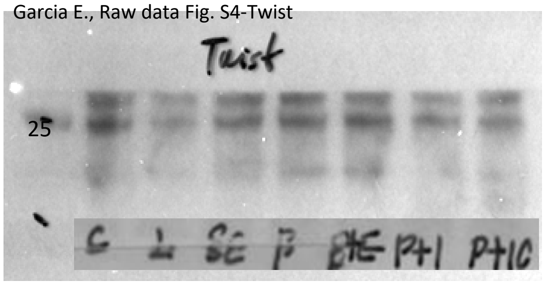

Twist 25KDa

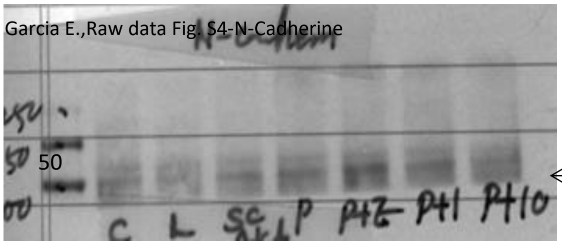

N-Cadherine 100KDa

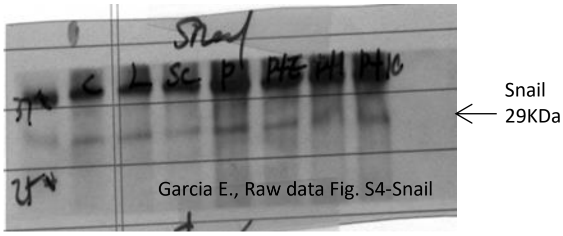

Snail 29KDa

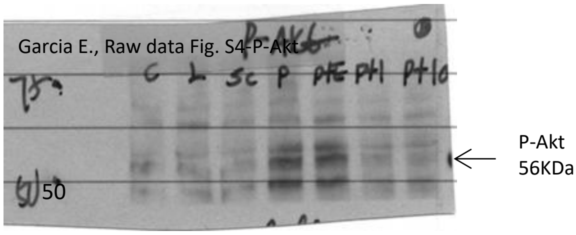

P-Akt 56KDa

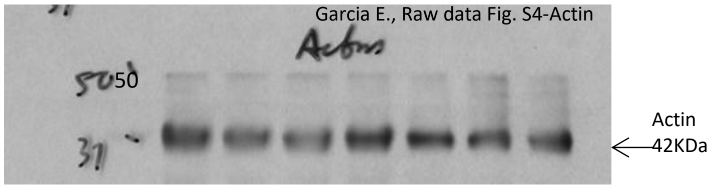

Actin 42KDa

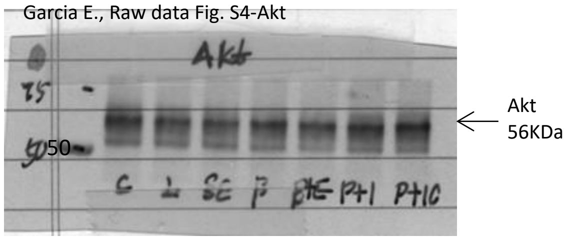

Akt 56KDa

Key:

C = Control (untreated)
L = Lipofectamine treated (Transfection control
Sc = Scrambled siRNA
P = PTEN SiRNA
P+E = P + Ethanol Vehicle
P+1 = P + 1 μM Nitrofen
P+10 = P + 10 μM Nitrofen

Garcia E., Raw data Fig. S4

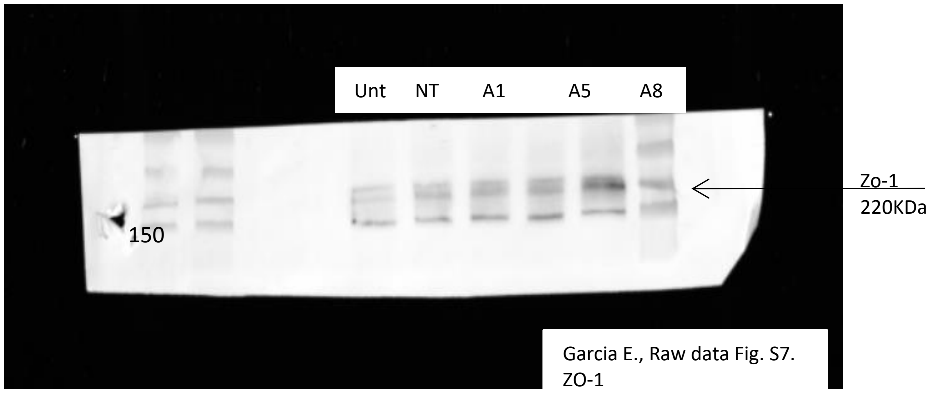

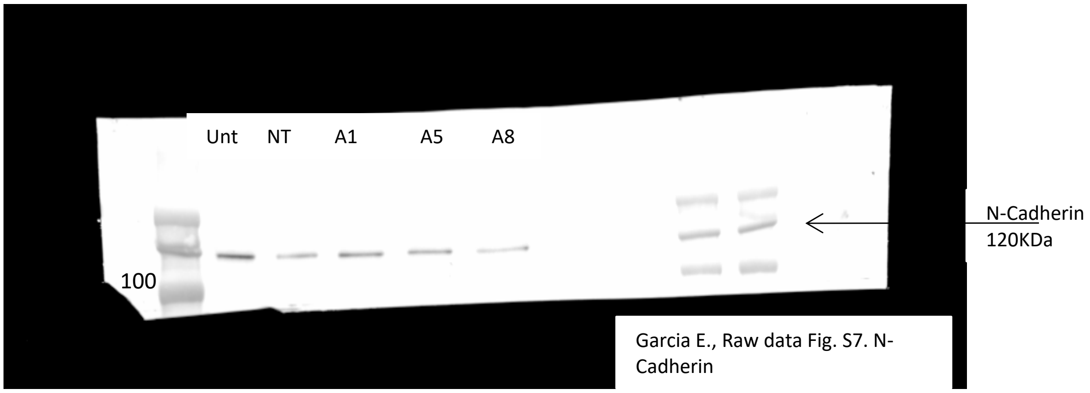

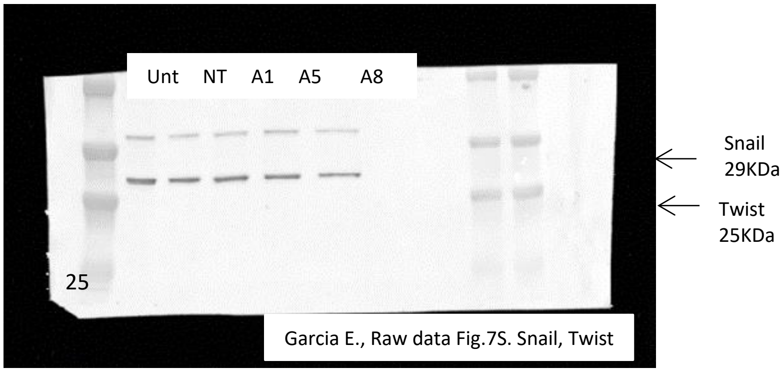

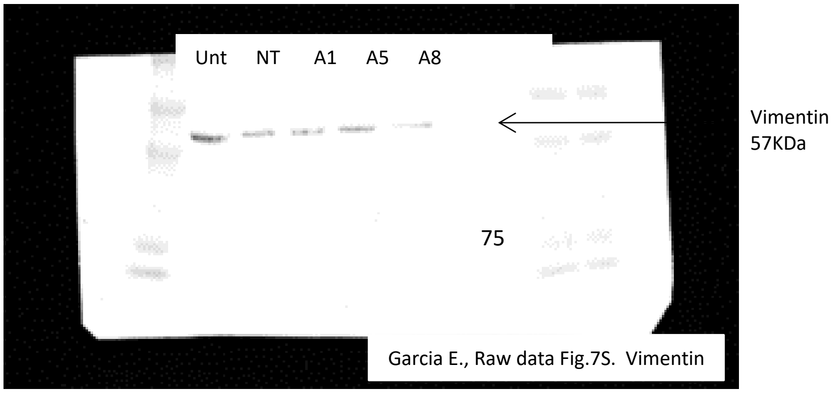

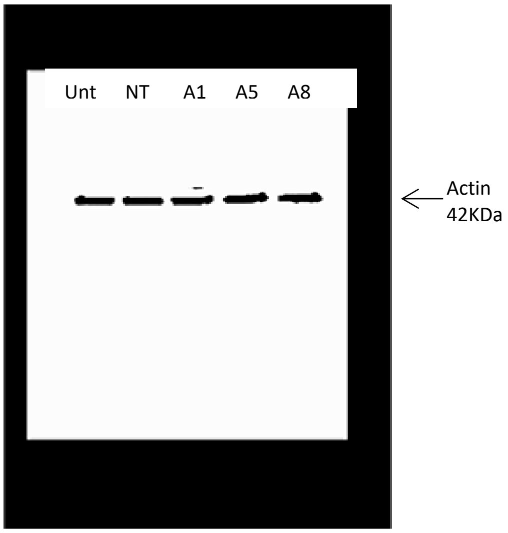

Garcia E., Raw data Fig. S7
